# Pressure-Induced Interlayer Bonding in Arsenic Revealed by Single-Crystal Synchrotron X‑ray Diffraction in a Diamond Anvil Cell

**DOI:** 10.1021/jacs.6c09191

**Published:** 2026-09-23

**Authors:** Matteo Ceppatelli, Marta Morana, Enrico Berretti, Kamil Dziubek, Konstantin Glazyrin, Demetrio Scelta, Alessandro Lavacchi, Roberto Bini, Maurizio Peruzzini, Manuel Serrano-Ruiz

**Affiliations:** † ICCOM-CNR, Institute of Chemistry of OrganoMetallic Compounds, National Research Council of Italy, Via Madonna del Piano 10, I-50019 Sesto Fiorentino, Firenze, Italy; ‡ LENS, 9300 European Laboratory for Non-linear Spectroscopy, Via N. Carrara 1, I-50019 Sesto Fiorentino, Firenze, Italy; § Dipartimento di Scienze Della Terra, 201843 Università degli Studi di Firenze, Via La Pira 4, I-50121 Firenze (FI), Italy; ∥ Department of Mineralogy and Crystallography, 27258 Universität Wien, Josef-Holaubek-Platz 2 (UZA II), 1090 Wien, Austria; ⊥ 28332 Deutsches Elektronen-Synchrotron DESY, Notkestr. 85, 22607 Hamburg, Germany; # Dipartimento di Chimica 226476 “Ugo Schiff”dell’Università degli Studi di Firenze, Via della Lastruccia 3, I-50019 Sesto Fiorentino, Firenze Italy

## Abstract

Arsenic was studied by single-crystal synchrotron X-ray diffraction during room *T* compression up to 37.8 GPa across the rhombohedral (As–I, A7) to simple cubic (As–II, sc) phase transition, using a diamond anvil cell for pressure generation and He as a pressure-transmitting medium. The pressure dependence of the rhombohedral lattice parameter *a*, angle α, atomic position parameter *u*, and first (*d*_1_) and second (*d*_2_) neighbor distances provides experimental evidence for a two-step pressure-induced interlayer bond formation mechanism, underlying the transition from the layered A7 structure to the nonlayered simple cubic one. Consistent with phosphorus, our data demonstrate the existence of an intermediate pseudo simple-cubic (p-sc) structure between 26.5 and 31.8 GPa. Within this pressure interval, where α = 60° and *u* < 0.250, the progressive reduction of the interlayer distances (*d*_1_) and the corresponding elongation of the intralayer ones (*d*_2_) reveal a pressure-induced *trans-*influence effect, underpinning the transition from trigonal pyramidal to octahedral atomic coordination and relating the formation of the p-sc structure to the persistence of *s-p* orbital mixing and stereochemically active lone pair electron density. While solving a long-standing structural issue in As, this study provides in-depth chemical insights concerning bond theory in pnictogens and the synthesis of innovative As-based materials.

## Graphical abstract

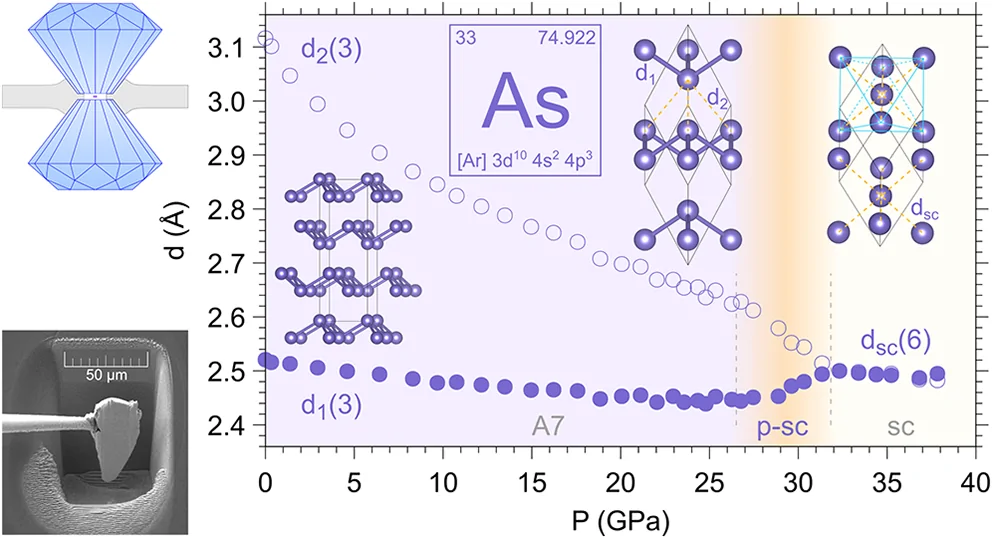

## Introduction

The high-pressure behavior of arsenic has been explored in the literature up to 250 GPa along the room *T* isotherm.^1^ At ambient pressure, the thermodynamically stable allotrope of As is the layered crystalline As–I structure (A7, *R*3̅*m*), commonly known as gray arsenic, which is isostructural to high-pressure A7 phosphorus and to ambient pressure Sb–I and Bi–I.^2,3^ Upon room *T* compression, As–I converts into simple cubic As–II (sc, *Pm*3̅*m*), with contrasting transition pressures experimentally reported at 25 GPa^4^ and between 31.4 and 36.6 GPa.^5^ On further compression, at ∼48 GPa, As–II sluggishly transforms into host–guest incommensurately modulated As–III,^6–8^ which has been observed to persist up to 94 GPa^6^ and 126 GPa,^1^ before ultimately converting into *bcc* As–IV.^1,6^

The phase transition from A7 As–I to sc As–II is particularly important for several aspects. While setting the high-pressure limit for the existence of layers in crystalline As, this phase transition represents indeed a long-debated prototypical transformation in structural chemistry. Particularly, it describes the pressure-induced transition from a semimetallic layered structure, in which As exhibits trigonal pyramidal coordination by three nearest neighbors in the same layer and hosts stereochemically active lone pair (LP) electron density (Figure 1A), to a metallic nonlayered structure, in which As is octahedrally coordinated by six equivalent nearest neighbors with no LP stereochemical activity (Figure 1B). From a chemical point of view, understanding the mechanism of the A7 to sc phase transition in As is paramount for chemical bond theory to model phenomena, including electron LP localization and interlayer bond formation, which represent key features to enable specific properties in systems exhibiting strong primary bonds and weak secondary interactions.^9–12^ Moreover, like in P, this phase transition in As is apparently related to the pressure dependence of the superconducting *T*_c_.^13,14^

**Figure 1:**
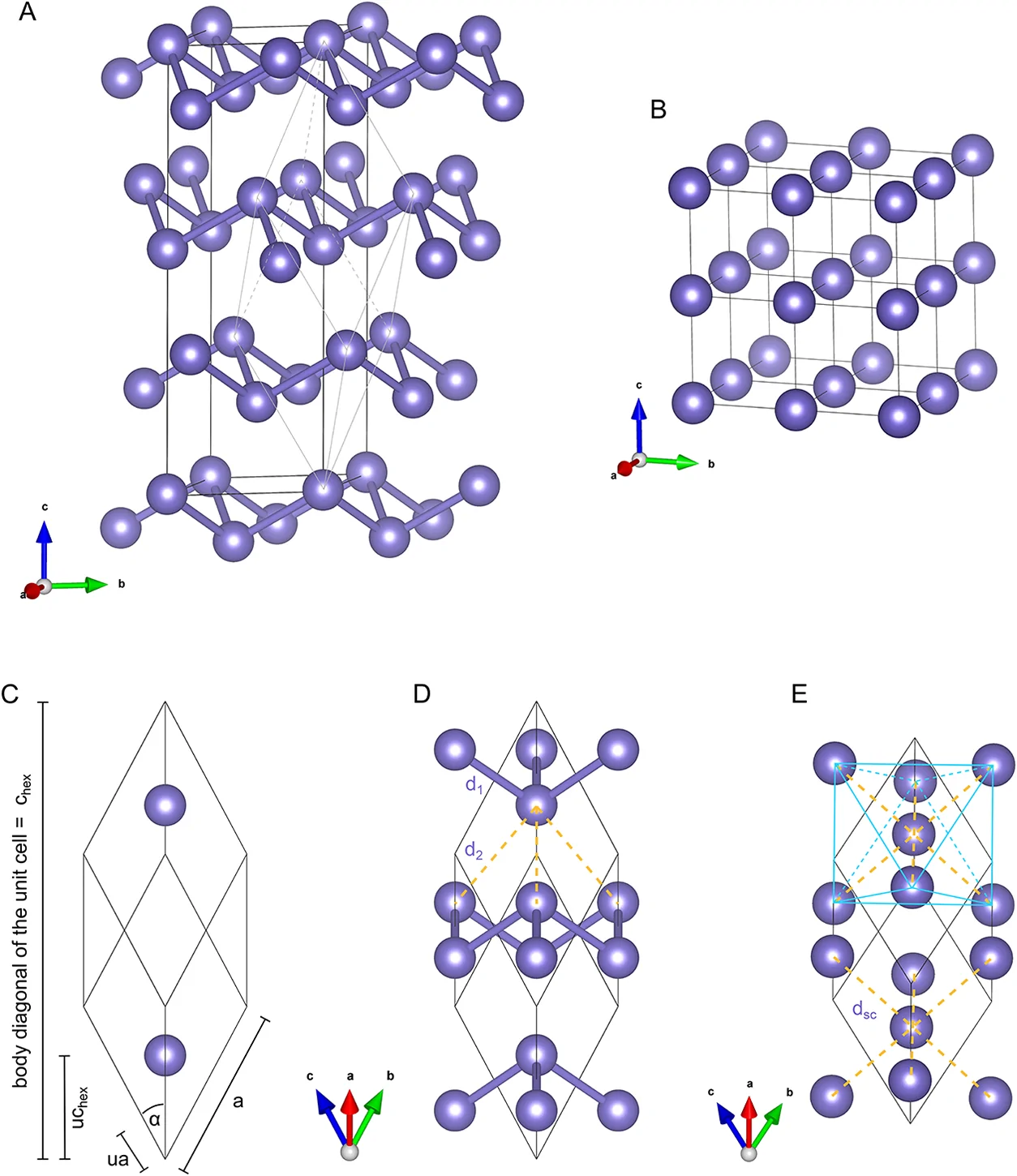
(A) Crystal structure and unit cell of A7 As–I (*R*3̅*m*) in hexagonal (*Z* = 6, black) and rhombohedral (*Z* = 2, gray) setting at ambient pressure. The presence of layers is highlighted by the intralayer covalent bonds connecting atoms. (B) Crystal structure and unit cell of sc As–II (*Pm*3̅*m*, *Z* = 1) at 32.3 GPa and 293 K. (C) Rhombohedral unit cell of As–I at ambient conditions, highlighting the angle α, the lattice parameter *a*, and the atomic position parameter *u*. The unit cell contains atoms at *u,u,u* and *u̅*, *u̅*, *u̅* aligned along the body diagonal. (D) First (*d*_1_) and second (*d*_2_) neighbor distances in As-I at ambient conditions, corresponding, respectively, to covalent intralayer bonds and weaker interlayer interactions (orange dashed lines), highlighting the trigonal-pyramidal coordination of As and the underlying LP electron density localization at the apex of the trigonal pyramid. (E) Crystal structure and rhombohedral unit cell of As–II at 32.3 GPa and 293 K (α = 60°, *u* = 0.250), highlighting the octahedral coordination (light-blue lines) of As with six equivalent nearest-neighbor distances (*d*_sc_), represented by orange dashed lines.

On a practical perspective, potential implications concern arsenene and in general pnictogen-based layered systems and thermoelectric, topological, and phase change materials.^15–20^

Nevertheless, after several decades of investigations, the transition pressure, the nature, and the mechanism of the A7 → sc phase transition in As remain elusive and still debated, essentially due to the lack of conclusive experimental data.^14,19,21,22^ Surprisingly, most of the articles attempting to model this phase transition in As are entirely grounded on the contrasting powder X-ray diffraction (XRD) data by two experimental studies dating back to a few decades,^4,5^ which have set the benchmark for any theoretical investigation so far.

Despite providing precious information, the XRD studies by Kikegawa and Iwasaki^5^ and by Beister et al.^4^ are affected by critical limitations essentially related to the lack of atomic position determination, to the use of a nonhydrostatic pressure-transmitting medium (PTM), to the insufficient pressure sampling resolution and investigated pressure range, and to the identification criterion for the phase transition, thereby making the fine structural details of this elusive transformation inaccessible. Particularly, as the nature of the sample is concerned, the inherent occurrence of heavy texturing in polycrystalline As samples makes powder-XRD ineffective to full structure determination (Section SI-1 in the Supporting Information).

On the theoretical side, various computational studies have modeled the A7 → sc phase transition in As.^13,14,21–31^ However, the small energy difference between the two structures and the rapid change in the density of states (DOS) across the Fermi level, requiring high levels of convergence, have represented a computational challenge for a long time, leading to calculated transition pressures scattered from 19 to 36 GPa^22^ (SI-1).

Recent studies, exploring the high-pressure behavior of gray^32^ and metastable black arsenic,^33,34^ locating the A7 → sc phase transition at 22 GPa (Raman),^33^ 25 GPa (XRD),^33^ 25.9 GPa (Raman),^34^ and 28 GPa (Raman),^32^ are affected by the same experimental limitations, with additional complications raised by the coexistence of different crystal structures, by the presence of defects and impurities and by the use of black-As flakes (SI-2).

To the best of our knowledge, no conclusive experimental structural data on this prototypical phase transition have been published.

Here, we report a synchrotron XRD study on the room *T* compression of single-crystal As in a diamond anvil cell (DAC) up to 37.8 GPa, using He as a PTM. Our experiments, enabling unambiguous high-pressure single-crystal structure determination, provide conclusive in-depth characterization of the pressure-induced interlayer bonding mechanism underlying the A7 to sc transformation, revealing the existence of an intermediate pseudo simple-cubic (p-sc) structure between A7 As–I and sc As–II, analogous to the p-sc structure observed in P,^35,36^ and highlighting the occurrence of *trans-*influence associated with the increase in As coordination. While finally addressing a long-standing open issue about As, our results demonstrate structural consistency in the high-pressure behavior of As compared with lighter P, highlighting the importance of orbital effects and LP electron density localization, which has recently emerged as a key feature in the high-pressure chemistry of group 15 elements.^37–43^

## Results and Discussion

The A7 structure of As–I belongs to the space group *R*3̅*m* (n. 166) in the trigonal crystal system and can be alternatively described in terms of hexagonal (*Z* = 6) or rhombohedral (*Z* = 2) settings (Figure 1A). The sc structure of As–II belongs to the higher symmetry space group *Pm*3̅*m* (n. 221) in the cubic crystal system (Figure 1B). However, the hexagonal or rhombohedral unit cell can still be used to describe sc As–II, while comparing specific structural parameters. For the purpose of this study, the adoption of the rhombohedral setting description for both As–I and As–II structures turns out to be particularly convenient, as it enables monitoring of the A7 to sc transformation throughout the entire process by the pressure dependence of the same quantities: the lattice parameter *a*, the angle α, the atomic position parameter *u*, and the first (*d*_1_, intralayer) and second (*d*_2_, interlayer) neighbor distances (Figure 1C–E). In the rhombohedral setting (*Z* = 2), the As atoms occupy 2*c* Wyckoff sites at *u,u,u* and *u̅*, *u̅*, *u̅* positions. The dimensionless atomic position parameter *u* represents the fractional atomic coordinate along each of the three axes and along the body diagonal of the unit cell. The sc structure corresponds to the special conditions defined by α = 60° and *u* = 0.250.

Our experiments were specifically designed and performed to address all the above-mentioned issues affecting previous studies and to catch the fine details of this elusive phase transition. All the steps of sample preparation, aimed at obtaining and preserving single-crystal quality throughout the whole process, from the synthesis of large high-purity As single crystals to the obtainment of small high-quality single crystals of suitable size for the DAC sample chamber by focused ion beam (FIB) processing, represented indeed critical challenges to the success of the experiments. Experimental details are highlighted in Figure 2 and extensively described in the SI.

**Figure 2:**
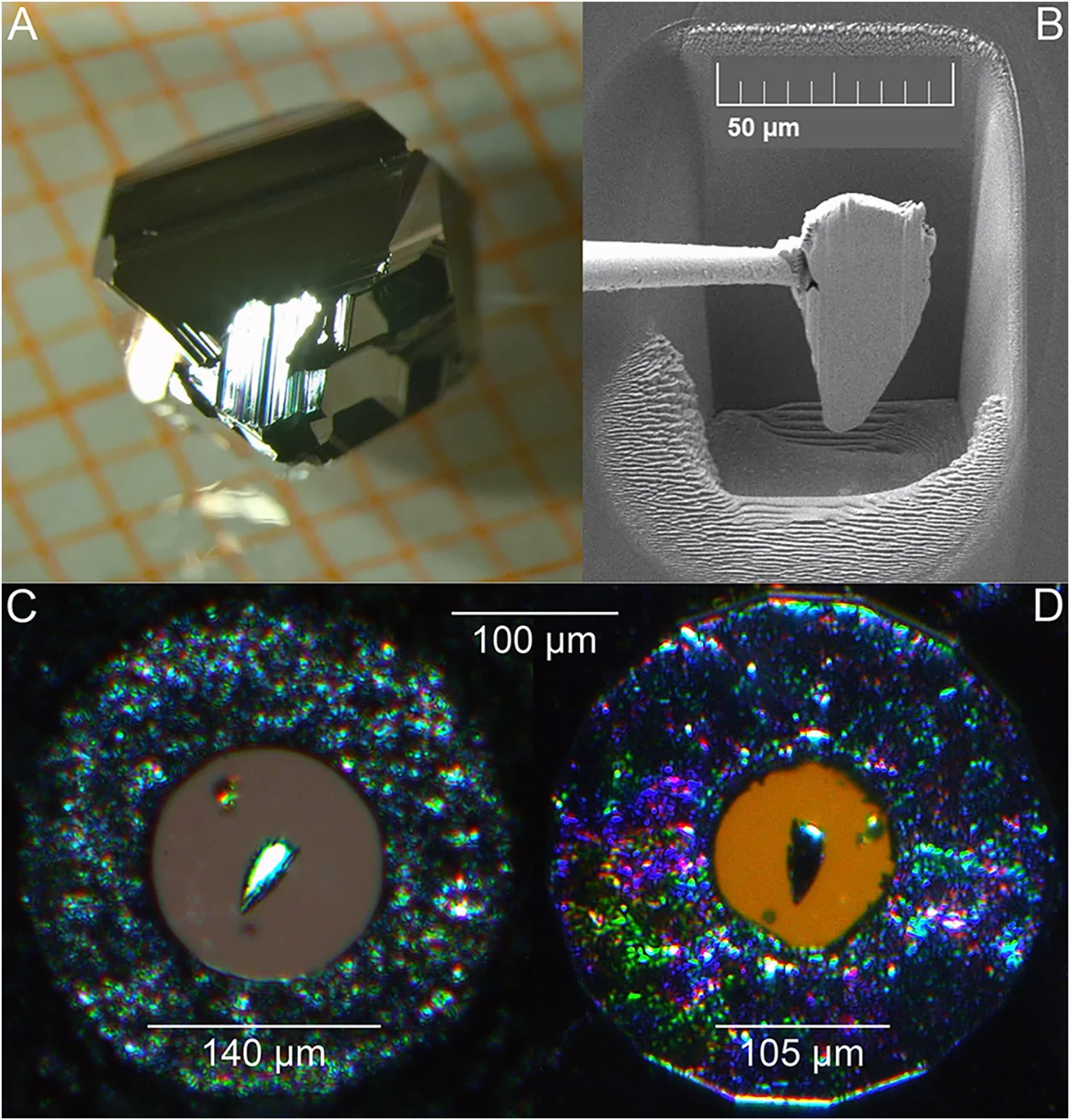
(A) Microscopy image of a mm^3^-sized single crystal of gray As after purification and recrystallization; (B) SEM image of a small single crystal of gray As in the shape of a trigonal prism (46 μm × 20 μm × 8 μm) obtained by FIB processing. (C) Photomicrograph of the small As single crystal inside the DAC at 0.1 GPa after gas-loading He and before starting the compression. The image was acquired from the side of the cell, where the As crystal was placed in contact with the diamond surface, as attested by the shiny reflecting surface of the crystal. (D) Photomicrograph of the recovered As single crystal inside the DAC at ambient pressure after room *T* compression up to 37.8 GPa and further decompression. The image was acquired immediately after releasing pressure at the end of the experiment from the opposite side of the cell compared to C. The gasket hole diameter in D is smaller than in C due to the gasket shrinkage upon compression. Based on the thickness of the As single crystal, specifically derived by FIB processing, and on the microscopic observation of the sample before, during and after compression, no sign of bridging by the diamond anvils was detected throughout the full pressure range of our study.

Single-crystal synchrotron XRD in DAC was performed at the P02.2 beamline (PETRA-III, DESY),^44^ under the best possible chemically inert and hydrostatic conditions ensured by the use of He as a PTM, and under high-resolution pressure sampling. Single-crystal structure determination enabled fine monitoring of the pressure dependence of *a*, α, *u*, *d*_1_, and *d*_2_ along the room *T* isotherm up to 37.8 GPa, providing direct insights into the elusive details of the A7 to sc phase transition.

Relevant refinement parameters and related errors are reported in Table SI-1. Data errors are typically smaller than the symbol size used in the figures. The Crystallographic Information Files (CIFs) of the solved crystal structures have been deposited in the CCDC-ICSD database under Deposition Numbers CSD 2546719–2546754 and are provided free of charge by the joint Cambridge Crystallographic Data Centre and Fachinformationszentrum Karlsruhe Access Structures service.

Our results are essentially condensed in Figure 3. In the stability range of the As–I structure, α and *u* smoothly increase with pressure. Correspondingly, due to the increase of density, *a*, *d*_1_, and *d*_2_ reduce. The faster decrease of *d*_2_ compared to *d*_1_ is consistent with the chemical bonding framework of layered A7 As–I, in which *d*_2_ corresponds to longer interlayer atomic distances ruled by weak interactions involving the electron LPs and *d*_1_ corresponds to shorter intralayer atomic distances, determined by covalent single bonds. This occurrence is particularly evident also by the pressure dependence of the *a*_hex_ and *c*_hex_ lattice parameters of the As–I structure described in hexagonal setting, which are indeed aligned parallel and perpendicular to the layers, respectively. The compressibility along *c*_hex_ is accordingly larger (Figure 4A and 4B). No discontinuity due to the solidification of He can be observed at 11.5 GPa.^45^

**Figure 3:**
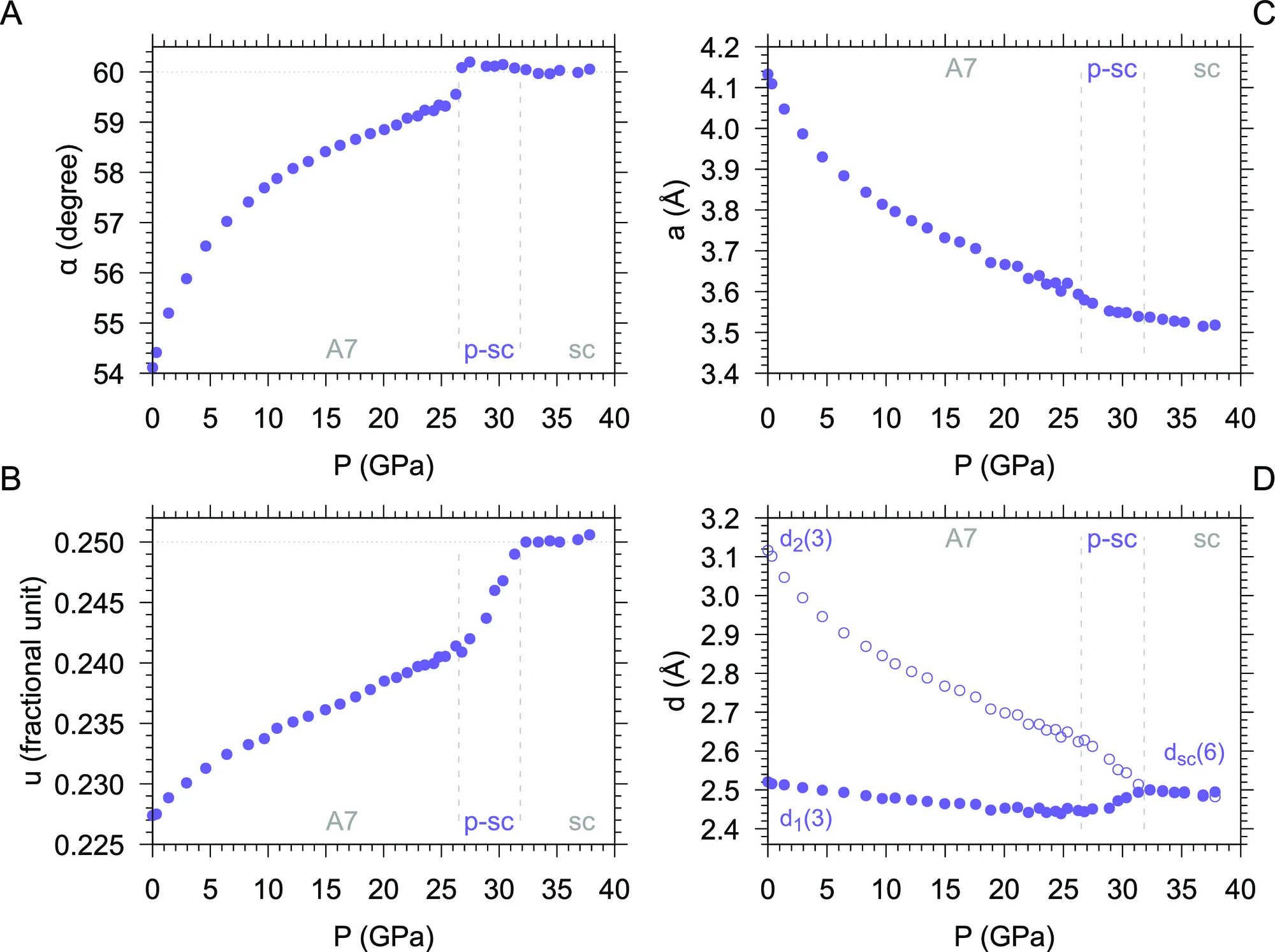
Pressure dependence of the angle α (A), atomic position parameter *u* (B), lattice parameter *a* (C), and first (*d*_1_, filled symbols) and second (*d*_2_, open symbols) neighbor distances (D) of the A7 As–I structure and equivalent interatomic distances (*d*_sc_) of the sc As–II one in the rhombohedral setting (*R*3̅*m*), across the A7 to sc transformation, through the intermediate p-sc structure. The vertical gray dashed lines mark the 26.5 and 31.8 GPa pressures at which α and *u*, respectively, reach 60° and 0.250 values, delimiting the pressure range of the p-sc structure. The horizontal gray dotted lines mark the limit values for α = 60.0° and *u* = 0.250 in the sc structure.

**Figure 4:**
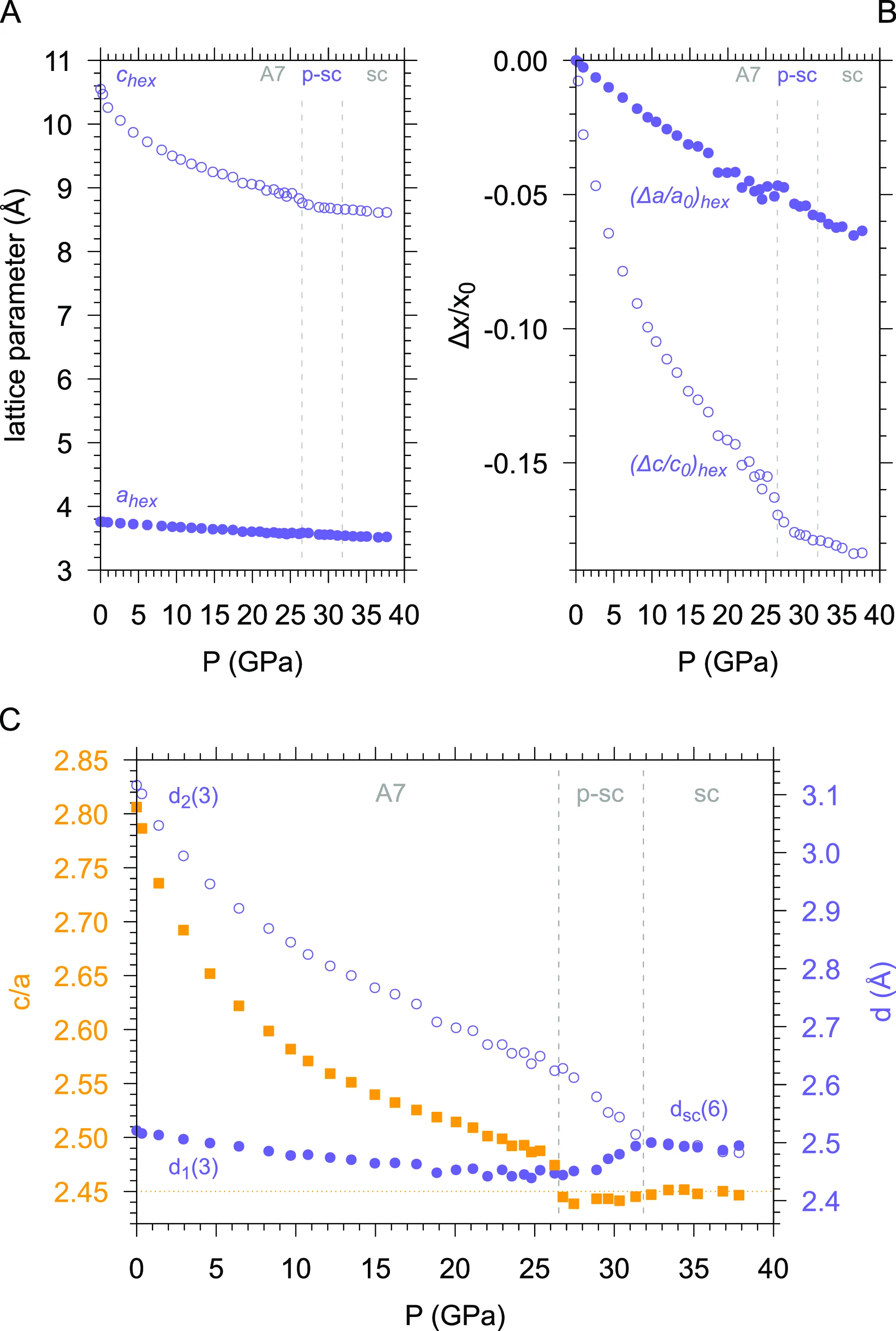
Pressure dependence of the *a*_hex_ and *c*_hex_ lattice parameters of the As–I (A7) and As–II (sc) structures described by the *R*3̅*m* space group in the hexagonal setting (A) and their relative variations with respect to the corresponding ambient pressure values ( $\frac{\Delta x}{x_{0}}$ , where Δ*x* = *x* – *x*_0_, *x* = *a*_hex_, *c*_hex_ and *x*_0_ = *a*_0,hex_, *c*_0,*h*ex_, with *a*_0,hex_ = 3.7599(2) Å and *c*_0,hex_ = 10.551(2) Å right). (B) Filled and open symbols, respectively, refer to the *a*_hex_ and *c*_hex_ lattice parameters. The vertical gray dashed lines mark the 26.5 and 31.8 GPa pressures at which α and *u* (rhombohedral setting), respectively, reach 60° and 0.250 values delimiting the pressure range of the p-sc structure. (C) Pressure dependence of the *c/a* hexagonal lattice parameters ratio (orange filled squares, left *y*-axis) and of the first *d*_1_ (purple filled circles, right *y*-axis) and second *d*_2_ (purple open circles, right *y*-axis) neighbor distances. The orange horizontal dotted line indicates the theoretical √6 = 2.45 value of the *c*/*a* ratio imposed by a cubic lattice achieved at 26.5 GPa, when α = 60°. The pressure range between 26.5 and 31.8 GPa, delimited by the gray vertical dashed lines and corresponding the p-sc structure (α = 60°, *u* < 0.250), highlights the *trans-*influence effect underlying the interlayer bond formation process, as evinced by the pressure dependence of *d*_1_ and *d*_2_, while the lattice is already cubic. Above 31.8 GPa, the three *d*_1_ and the three *d*_2_ distances merge into six equivalent *d*_*sc*_ distances, indicating the attainment of the sc structure.

Noticeably, a slight slope variation can be appreciated at 8–10 GPa in the pressure dependence of α, *u*, *a*, *d*_1_ and *d*_2_ (Figure 3).

However, on further compression, our single-crystal data reveal new structural and chemical insights compared to previous studies. As a matter of fact, our data clearly show that α and *u*, respectively, reach their 60° and 0.250 values, characterizing the sc structure, at different pressures. While at 26.5 GPa, α suddenly jumps to 60°, without changing anymore on further compression, at the same pressure, *u* = 0.241 is still lower than the 0.250 value of sc As–II, which is only reached on further compression at 31.8 GPa (Figures 3A, 3B, and SI-3). Furthermore, while *a* smoothly decreases with pressure, showing only slight slope variations at 26.5 and 31.8 GPa, the pressure dependence of *d*_1_ and *d*_2_ exhibits instead remarkable and highly informative changes at 26.5 GPa, when α = 60° (Figure 3). Indeed, while the slope of *d*_2_ becomes more negative, indicating faster shortening of the three interlayer distances, the slope of *d*_1_ becomes positive, revealing an elongation of the three intralayer ones, until *d*_1_ and *d*_2_ converge into six equivalent interatomic distances (*d*_sc_) at 31.8 GPa, as expected in the sc structure. From an atomic coordination point of view, this experimental evidence marks the transition from a layered structure with trigonal pyramidal coordination to a nonlayered structure with octahedral coordination. Noticeably, the pressure value, at which *d*_1_ and *d*_2_ are observed to merge into *d*_sc_, corresponds to the attainment of the 0.250 value by the atomic position *u*. Above 31.8 GPa and up to the highest investigated pressure, *d*_sc_ slightly and continuously decreases with pressure, consistent with the expected stability of the sc structure.

Our data demonstrate that the A7 to sc phase transition is a two-step process. In the first step, taking place at 26.5 GPa, α suddenly reaches the 60° value expected in the sc structure, while *u* = 0.241 still remains significantly lower than the 0.250 sc value (Figures 3A, 3B, and SI-3). From a structural point of view, this occurrence indicates a first-order rhombohedral to simple cubic lattice transition, in which the atoms have not yet moved to the expected positions of the simple cubic structure. In the second step, taking place on further compression above 26.5 GPa and culminating at 31.8 GPa, while α remains constantly equal to 60°, the atomic position parameter *u* increases faster, progressively reaching the 0.250 value expected in the sc structure at 31.8 GPa and thereafter remaining constant up to the highest investigated pressure (Figures 3A, 3B, and SI-3). This occurrence, indicating the continuous displacement of the atoms toward the sc sites, corresponds to the attainment of the full symmetry of the sc structure (*Pm*3̅*m*), through a second-order phase transition^46^ (SI-7).

Overall, the identification of a pressure interval, in which α = 60° and *u* < 0.250, reveals the existence of a pseudo simple-cubic (p-sc) structure (*R*3̅*m*) in the phase diagram of As, intermediate between layered A7 As–I and nonlayered sc As–II.

The transformation of the rhombohedral lattice into a simple cubic one during the first step of the transformation is even more evident from the pressure dependence of the *c/a* hexagonal lattice parameter ratio, suddenly reaching the theoretical 2.45 value for a cubic lattice at 26.5 GPa, when α becomes 60° (Figure 4C).^4,5,35,36^ In this regard, the separation in pressure of the second step, involving the atomic displacement toward the sc sites, clearly emerges when comparing the pressure dependence of *c/a* with that of *d*_1_ and *d*_2_ (Figure 4C). Indeed, at 26.5 GPa, when α = 60° and *c/a* = 2.45, the three interlayer atomic distances *d*_2_ begin to shorten faster with pressure, as attested by the slope of *d*_2_ becoming more negative, indicating the strengthening of the corresponding secondary interactions. Remarkably, at the same pressure, the three intralayer atomic distances *d*_1_ begin to elongate upon compression, as attested by the slope of *d*_1_ turning to positive, indicating the weakening of the corresponding covalent bonds. The shortening *d*_2_ and the elongating *d*_1_ distances finally merge into six equivalent interatomic distances at 31.8 GPa.

This phenomenon is known in inorganic chemistry as *trans-*influence of the weak secondary interactions on the strong primary bonds and is a typical scenario observed during multicenter bond formation,^9,10^ thus suggesting a similar mechanism to underlie the interlayer bond formation process characterizing the phase transition from A7 As–I to sc As–II and the related increase in the As coordination number from 3 to 6. Noticeably, the pressure range of the *trans-*influence corresponds to the pressure range of the p-sc structure, indicating that the two occurrences are intimately related.

The existence of the p-sc structure between 26.5 and 31.8 GPa is further confirmed by the observation of characteristic reflections in this pressure interval, consistently emerging in the p-sc structure, but not expected in the sc one. Despite the sample being a single crystal and Bragg peaks appearing as spots rather than rings, the integrated diffraction patterns shown in Figure 5 qualitatively highlight this occurrence. While the merging of the As peaks is completed between 26.5 and 27.5 GPa, weak reflections originating from this merging, which are not expected in the sc structure, still persist above this pressure. Their intensity progressively weakens on compression above 26.5 GPa, until completely vanishing when the full *Pm*3̅*m* symmetry of the sc structure is attained at 31.8 GPa. The pressure interval, where the weak reflections are observed to persist, corresponds to the pressure range of the p-sc structure, consistent with the behavior of the p-sc structure in P.^35,36^

**Figure 5:**
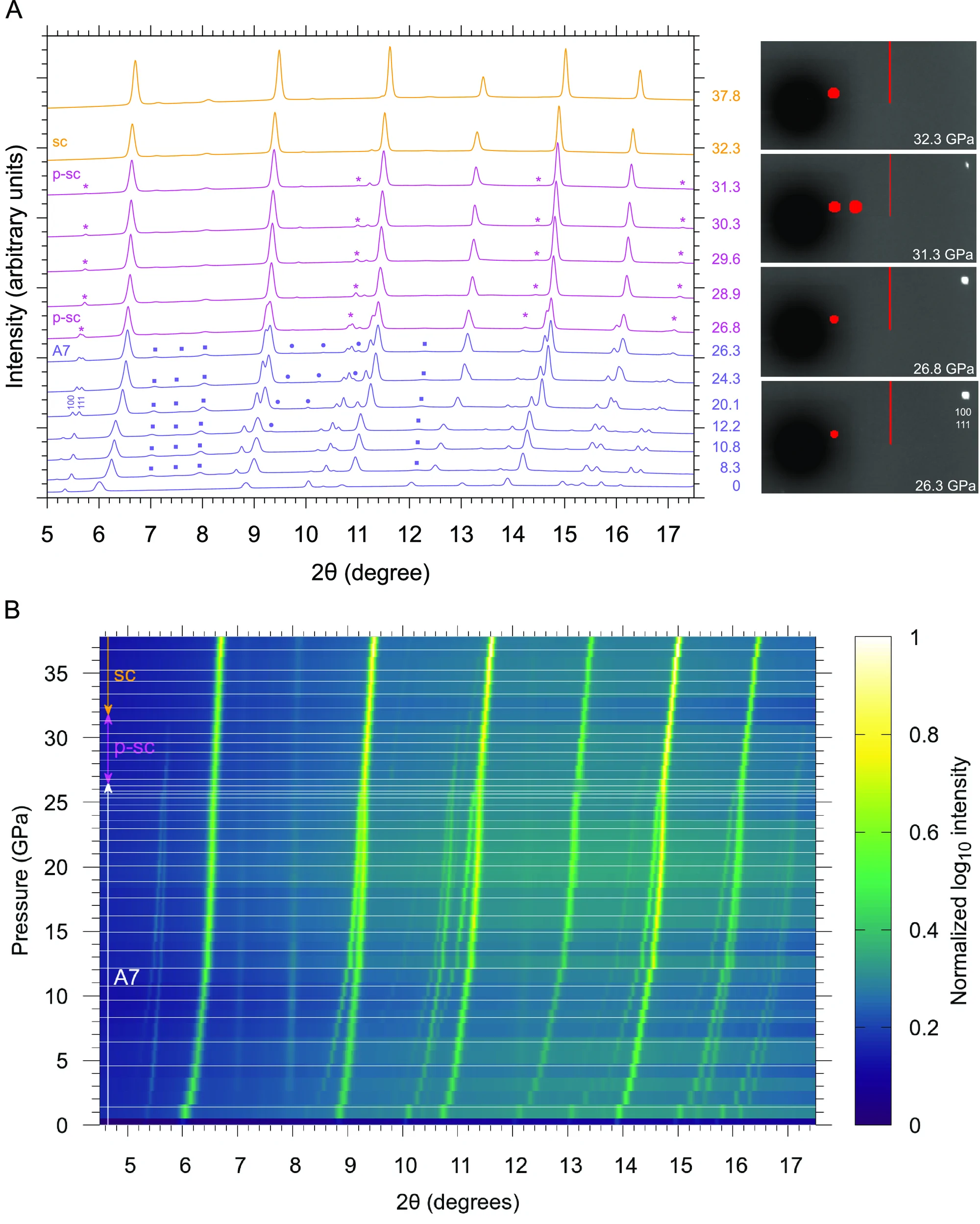
(A) Evolution with pressure of selected 1D integrated XRD patterns (λ = 0.2897 Å, ± 20° DAC oscillation) of As in He corresponding to the A7 (purple), p-sc (magenta), and sc (orange) structures. Contributions from the Re gasket (squares) and He (circles) after crystallization are also shown. The merging of the A7 peaks is completed at ∼27.5 GPa and the persistence of weak reflections (asterisks) indicates the formation of the p-sc structure. The intensity of these reflections, which are not expected in the pattern of sc As–II, progressively weakens until completely vanishing once the full *Pm*3̅*m* symmetry of the sc structure is attained. The detector images highlight this behavior for the low-angle 100 and 111 reflections (rhombohedral setting). The red areas mask diamond peaks and detector features. (B) 2D intensity color map as a function of the 2θ scattering angle and pressure, highlighting the merging of the A7 reflections, the persistence of the p-sc reflections between 26.5 and 31.8 GPa, and their disappearance at 32.3 GPa, with the attainment of the sc structure.

Overall, considering a slight difference in the absolute pressure values, at which α and *u* reach their limit values, and in the width of the pressure interval separating these two occurrences, our experimental results are in excellent agreement with the pressure dependence of α, *u*, *a*, *d*_1_ and *d*_2_ calculated by Silas et al. using DFT with GGA-PBE approximation^21^ (Figure 6). Silas et al. were somehow unsure whether their finding of α and *u*, respectively, reaching their limit values at different pressures (α = 60° at 25 GPa and *u* = 0.25 at 28 GPa) was due to a computational artifact deriving from the use of smearing of the cutoff energy in their calculations. However, our experiments provide evidence of this occurrence and indicate that the corresponding pressure interval (5.6 GPa) is actually almost twice as large as the one predicted by Silas et al. (3 GPa).^21^

**Figure 6:**
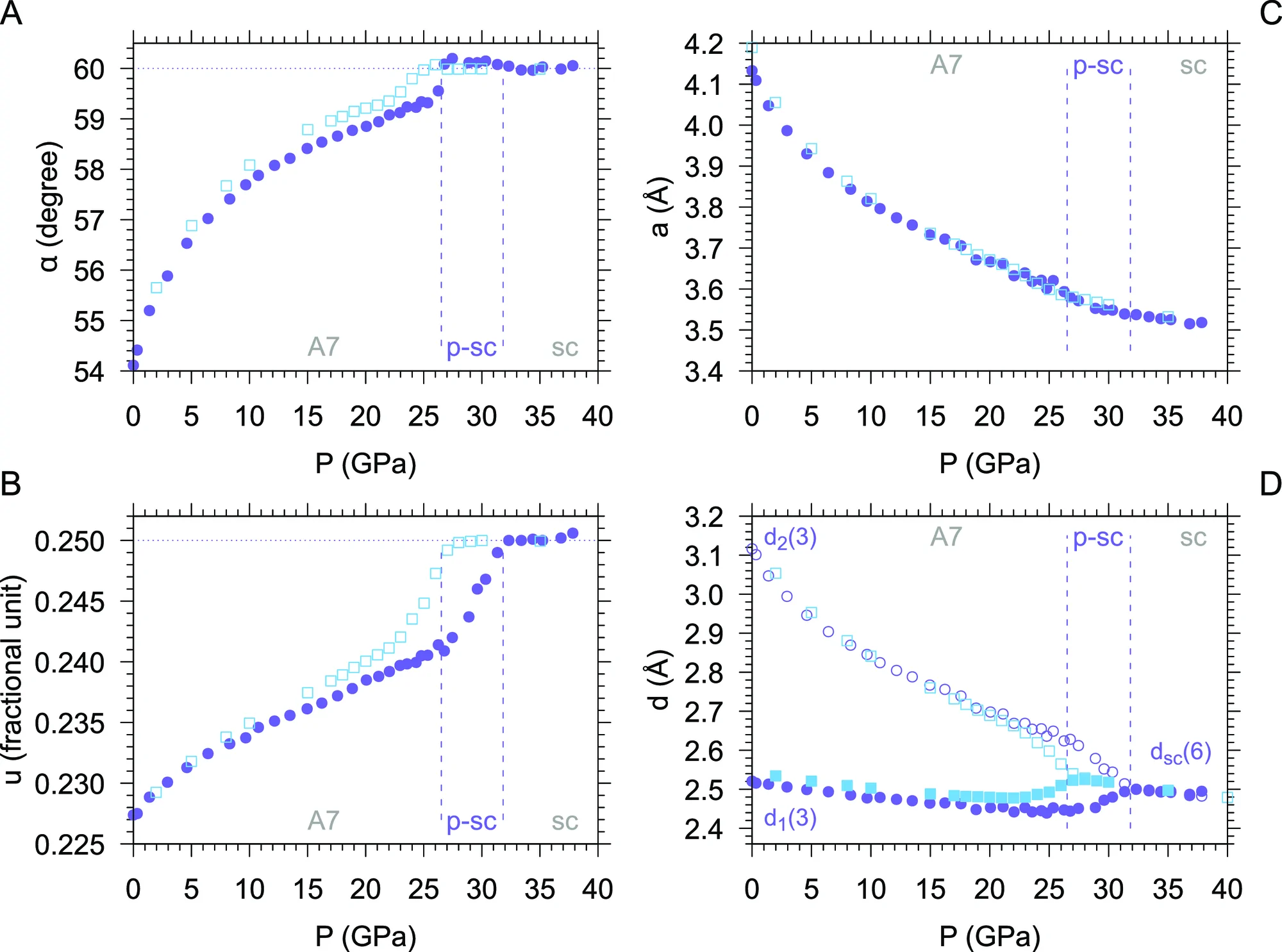
Comparison of the pressure dependence of experimental and theoretical values of the angle α (A), atomic position parameter *u* (B), lattice parameter *a* (C), and first (*d*_1_, filled symbols) and second (*d*_2_, open symbols) neighbor distances (D) of the As–I (A7) and As–II (sc) structures in the rhombohedral setting (*R*3̅*m*) across the A7 to sc transformation, through the intermediate p-sc structure discovered in this study (A7 → p-sc → sc). The vertical purple dashed lines mark the experimental pressure values of 26.5 and 31.8 GPa, at which α and *u*, respectively, reach the 60° and 0.250 values, delimiting the pressure range of the p-sc structure. The horizontal purple dotted lines mark the limit values of α = 60.0° and *u* = 0.250, indicating the attainment of the sc structure. Purple circles represent experimental data from this study, whereas light-blue squares represent calculated values by Silas et al. using the GGA-PBE approximation.^21^

It is worth noticing that although it is not mentioned in their articles,^21,22^ a possible alternative explanation for this occurrence, related to the existence of a different intermediate As structure between A7 As–I and sc As–II, was elsewhere suggested by Silas et al. to account for the mismatch in their calculated pressure dependence of α and *u*.^47^ Interestingly, a similar hypothesis was proposed by Chan et al. in the case of P,^48^ before the experimental discovery of the p-sc structure in the phase diagram of this element.^35,36^

The pressure-induced mechanism of interlayer bond formation underlying the A7 to sc transformation, through the intermediate p-sc structure, is indeed analogous to that observed in lighter P (A7 → p-sc → sc).^35,36^ Compared to P, where the p-sc structure appears at 10.5 GPa and persists up to at least ∼30 GPa, the p-sc structure in As forms at 26.5 GPa and extends over a much narrower pressure interval (5.6 GPa) up to 31.8 GPa. Noticeably, regarding the first step, while a sharp discontinuity of α at the A7 → p-sc transition is observed both in As (26.5 GPa) and P (10.5 GPa),^35^ the pressure dependence of *u* in As does not exhibit the discontinuity observed in P. Regarding the second step, the experimental results on As are particularly meaningful, providing the full pressure dependence of *u*, which in the case of P could not be conclusively ascertained.

Compared to the previous literature,^1,4–6,8,13,32–34,49,50^ where the A7 structure was thought to directly transform into the sc one, the discovery of the intermediate p-sc structure in As, along with the observation of the *trans-*influence, not only redefines its phase diagram but also provides new chemical insights into the mechanism of interlayer bond formation, while adding focus on the orbital interactions underlying this process (SI-7). In this regard, the discovery of the p-sc structure in As can be indeed accounted for in terms of competing pressure-dependent orbital effects and electrostatic interactions.^27,51–56^

Experimentally, the p-sc structure still exhibits three short intralayer *d*_1_ and three long interlayer *d*_2_ distances, rather than six equivalent ones, as instead expected in the sc structure. The p-sc structure is thus still a layered structure related to A7 As–I, featuring trigonal pyramidal rather than octahedral atomic coordination. This occurrence underlies the persistence of LP electron density localization and stereochemical activity, that are fingerprints of *s-p* orbital mixing. Theoretically, the A7 structure has been traditionally interpreted as a Peierls distortion of the sc structure and the A7 → sc phase transition has been accordingly pictured as a pressure-induced suppression of this Peierls distortion.^4,25,57–59^ However, the presence of *s-p* orbital mixing has been shown to play a role in stabilizing the A7 structure of P and suggested to be relevant also in As.^27,53,55,56,60,61^ Specifically, *s-p* orbital mixing is found to dominate at low pressure and to stabilize the A7 structure, whereas *s-d* orbital mixing, along with the electrostatic contribution, is found to take over on further compression and to stabilize the more compact sc structure at higher pressure, consistent with the increased contribution of the electron density of states at the Fermi level upon the phase transition.^27,53–56^

According to our interpretation, in the intermediate pressure range, a delicate interplay, between *s-p* orbital mixing on one side and *s-d* orbital mixing and electrostatic interactions on the other, is responsible for the existence of the p-sc structure. In these conditions, the lattice is cubic, but the displacement of the atoms toward the sc sites is hindered by the persistence of residual stereochemically active LP electron density, which is stabilized by *s-p* orbital mixing. LP electron expression and stereochemical activity, however, progressively vanish during the interlayer bond formation process, as *s-p* orbital mixing is reduced and *s-d* orbital mixing and electrostatic interactions become dominant with increasing pressure. The attainment of the sc structure is therefore impeded as long as this delicate interplay persists, suggesting the effective atomic orbital overlap characterizing the sc structure to be prevented by the persistence of *s-p* orbital mixing. Overall, the process reveals a progressive change from coordination number 3 in trigonal pyramidal coordination to coordination number 6 in octahedral coordination, in agreement with the pressure coordination rule.^62–64^

This interpretation also accounts for the differences in the high-pressure behavior of the p-sc structure in As compared with that in P, actually strengthening their consistency. As a matter of fact, the smaller pressure interval of the p-sc structure in As compared to P is consistent with the reduced extent of the *s-p* orbital mixing with increasing atomic number and with increasing pressure, whereas the onset of the p-sc structure in P at lower pressure compared to As can be accounted for by the fact that the *s-d* orbital mixing in P increases faster with pressure and is more effective than in As^53,56,61^ due to the presence of the empty 3*d* shell and to the specific pressure dependence of *d* orbitals in P^27,55,65,66^ (SI-8).

The progressive loss of LP electron density localization during the A7 to sc phase transition, through the intermediate p-sc structure (A7 → p-sc → sc), has been modeled for the analogous transformation in P.^67^ Specifically, Galy and Vignoles reported the gradual pressure-induced splitting of the LP electron density characterizing the A7 structure of P into a three-lobe orbital interaction with the three second neighbors during interlayer bond formation toward the sc structure.^35,36,67^ Interestingly, a similar orbital behavior has been recently modeled by Osman et al. for the A7 → sc phase transition in As.^19^ Despite not reporting any intermediate structure and calculating the phase transition to sharply take place at 25 GPa, Osman et al. observed a residual trigonal distortion of the sc structure in their calculations just above this pressure. Moreover, their calculated interatomic distances are in qualitative agreement with our experimental values and with the calculated values by Silas et al.,^21^ thus further supporting the occurrence of *trans-*influence and multicenter bonding.

Additionally, in their first study, Silas et al. correctly predicted α to reach its high-symmetry value of 60° at lower pressure compared to *u*,^21^ proposing the pressure-induced electronic changes associated with the A7 → sc phase transition to drive *u* to the 0.250 high-symmetry value. However, in their successive calculations on the pressure dependence of the Fermi surface, they suggested these electronic changes to rather first drive α to its high-symmetry value.^22^ This picture is fully consistent with our experimental data and further support the key role of orbital interactions and LP electron density localization in driving the mechanism of the A7 to sc phase transition and in determining the existence of the p-sc structure.

The persistence of *s-p* orbital mixing and the stereochemically active role of the LP electron density in determining the existence of the p-sc structure between 26.5 and 31.8 GPa are also highlighted by the pressure dependence of φ_1_, φ_2_, and φ_3_, respectively, representing the intralayer As–As–As angle of the trigonal pyramidal As coordination (φ_1_) and the interlayer equatorial-to-axial (φ_2_) and axial-to-axial (φ_3_) angles of the incipient octahedral coordination (Figure 7).

**Figure 7:**
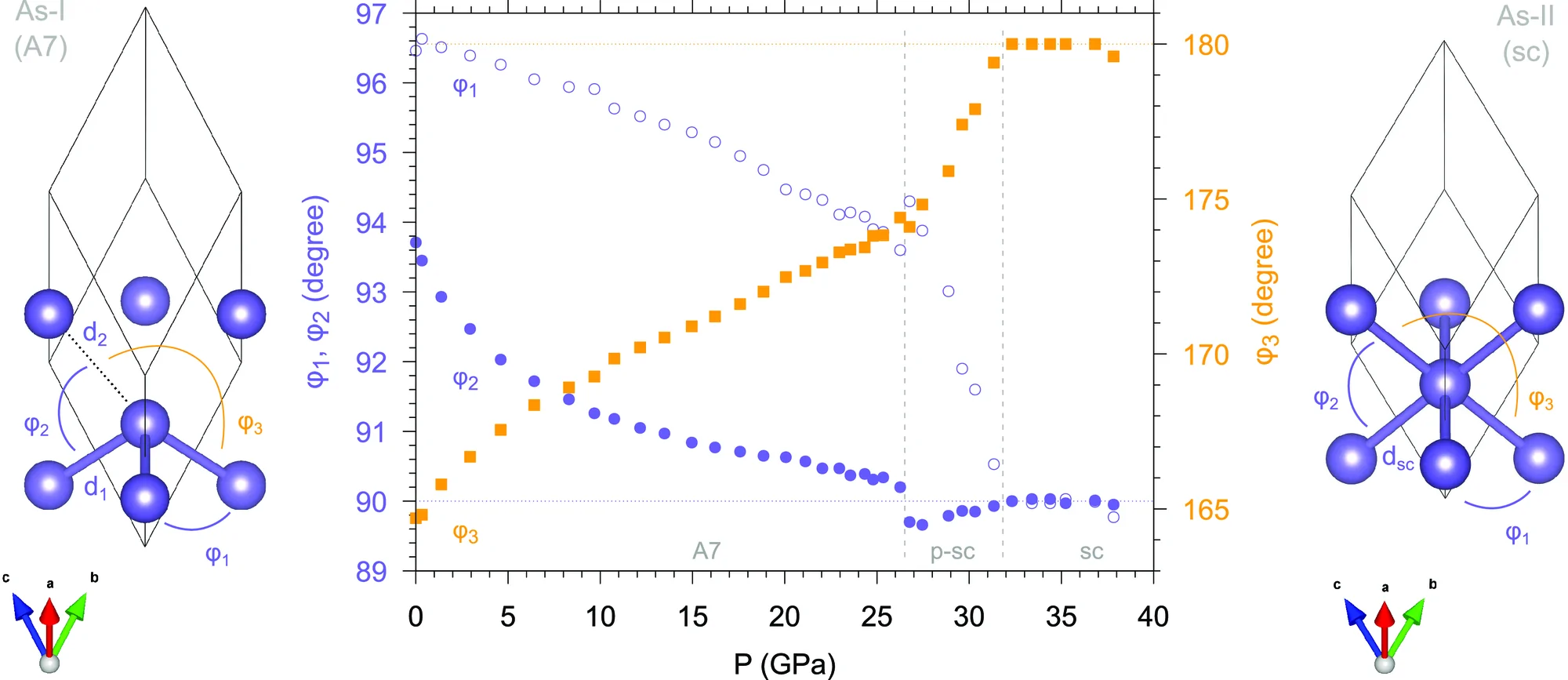
Pressure dependence of φ_1_, φ_2_, and φ_3_, representing, respectively, the intralayer As–As–As angle of the trigonal-pyramidal coordination (purple open circles, left *y*-axis values) and the interlayer equatorial-to-axial (purple filled circles, left *y*-axis values) and axial-to-axial (orange filled squares, right *y*-axis values) As–As–As angles of the incipient octahedral coordination, where φ_1_ = φ_2_ = 90° and φ_3_ = 180°. The 90° limit value for φ_1_ and φ_2_ and the 180° limit value for φ_3_ are marked by the purple and orange dotted lines, respectively, referring to the left and right *y*-axes. The gray vertical dashed lines mark the pressure values of 26.5 and 31.8 GPa, at which α and *u*, respectively, reach the 60° and 0.250 values. The two portions of the unit cells on the left- and right-hand sides, respectively, represent an As atom in trigonal pyramidal coordination, with three first neighbors at distance *d*_1_ and three second neighbors at distance *d*_2_ (*d*_1_ < *d*_2_) in the A7 structure of As–I at ambient pressure, and an As atom in octahedral coordination with six nearest neighbors at equivalent distance *d*_sc_ in the sc structure of As–II at 32.3 GPa.

While the three angles smoothly evolve with pressure in the A7 structure, φ_2_ suddenly approaches the 90° value at 26.5 GPa, when α = 60°. Correspondingly, at the same pressure, the pressure dependence of φ_1_ and φ_3_ exhibits an abrupt slope change. Between 26.5 and 31.8 GPa, in the pressure range of the p-sc structure, the abrupt decrease of the intralayer angle φ_1_ and the steep increase of the interlayer axial-to-axial one φ_3_, respectively, reaching their 90° and 180° sc values, mirror the *trans-*influence of the interatomic distances *d*_1_ and *d*_2_.

Based on the valence shell electron pair repulsion (VSEPR) theory, bonding angles resulting from local atomic orbital hybridization are particularly insightful at revealing LP stereochemical activity.^68^ The LP electron density at the apex of the trigonal pyramidal As coordination polyhedron, pointing at the adjacent layer and occupying the space between the three second neighbors, is expected to be directly involved in this process and to be particularly affected by the displacement of the atoms along the body cell diagonal, taking place in the second step of the transformation and leading to the final reduction of the interlayer distance. This interpretation consistently accounts for the abrupt changes in the pressure dependence of φ_1_ and φ_3_, underpinning the persistence of LP stereochemical activity and *s-p* orbital mixing in the pressure range of the p-sc structure. The attainment of the sc structure at 31.8 GPa, associated with the loss of LP stereochemical activity, is highlighted by the fulfillment of the φ_1_ = 90°, φ_2_ = 90°, and φ_3_ = 180° conditions.

Mirroring the evolution with the pressure of the *a* lattice parameter, the pressure dependence of the atomic volume does not show any clear discontinuity, in agreement with literature experimental data (Figure 8). In fact, the atomic volume variations across the A7 → p-sc and p-sc → sc phase transitions are very small (Δ*V*_*A*→p‑sc_^atom^ = 0.00 ± 0.05 Å^3^, Δ*V*_p‑sc→sc_^atom^ = −0.035 ± 0.017 Å^3^, Table SI-1), remarking the smooth and progressive rearrangement of the local atomic environment during the *trans-*influence effect, leading to the increase of the As coordination number from 3 to 6.

**Figure 8:**
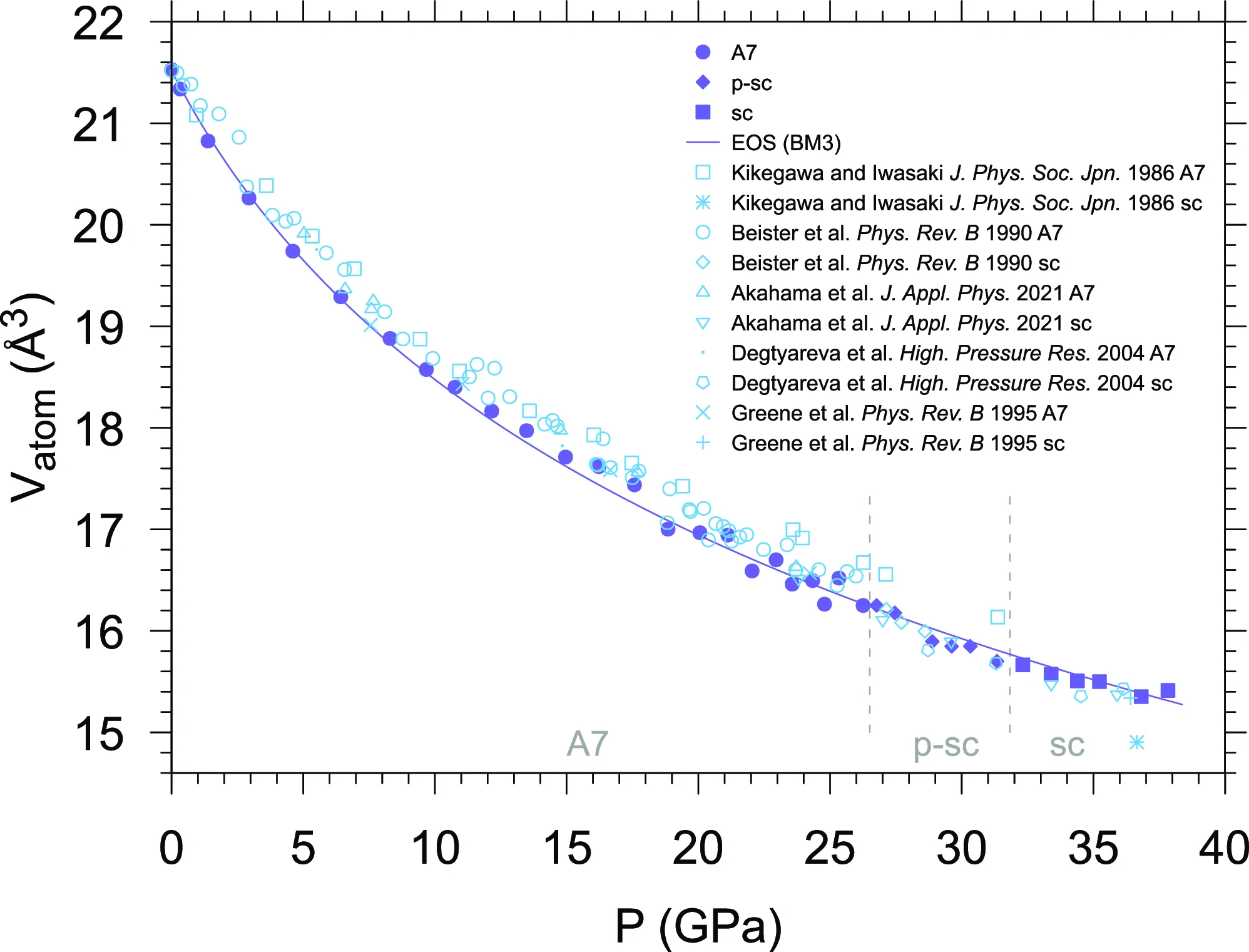
Pressure dependence of the atomic volume of As from this study (purple filled symbols: A7 circles, p-sc diamonds, sc squares) compared with experimental literature data (light-blue symbols): open squares (A7) and asterisks (sc),^5^ open circles (A7) and diamonds (sc),^4^ up (A7) and down (sc) triangles,^1^ dots (A7) and pentagons (sc),^8^ and crosses (A7) and pluses (sc).^6^ The vertical dashed lines mark the experimental pressure values of 26.5 and 31.8 GPa, corresponding to α = 60° and *u* = 0.250, respectively, and delimiting the pressure range of the p-sc structure in As. The solid line represents the third-order Birch–Murnaghan equation of state (BM3) fitting our complete dataset from ambient pressure up to 37.8 GPa (*B*_0_ = 42.6 ± 1.1 GPa, *B*_0_^′^ = 5.7 ± 0.2, *V*_0_^atom^ = 21.518 ± 0.008 Å^3^).

The equation of state (EOS) of As in the pressure range of the A7 and sc structures has been determined in the literature either by considering the A7 ^4,5^ and sc^1^ structures separately, thus deriving distinct EOS parameters for each phase, or by considering together the pressure dependence of their volumes.^6,8^ The latter procedure is somehow justified by the very small volume variation associated with the A7 to sc transformation and by the small pressure intervals for the existence of the two structures.

In Table 1, the bulk modulus *B*_0_, its first derivative *B*_0_^′^ and the atomic volume *V*_0_^atom^ at zero pressure, derived for the entire investigated pressure range across the A7, p-sc and sc structures (0–37.8 GPa) and for the pressure intervals delimiting the A7 (0–26.5 GPa), p-sc (26.5–31.8 GPa), A7 + p-sc (0–31.8 GPa), and p-sc + sc (26.5–37.8 GPa) structures, are compared with experimental and calculated values from the literature. No EOS parameters were derived for the sc structure due to the limited number of data points compared to its stability range. Additionally, due to the missing ambient pressure volume of the high-pressure phases of As, the EOS parameters listed in Table 1 for the p-sc and p-sc + sc intervals have been obtained by fixing *V*_0_^atom^ to our experimentally determined atomic volume of A7 As–I at ambient pressure or to the zero pressure atomic volume of the sc structure derived by the fit of high-pressure experiments^1^ and by calculations,^21^ as reported in the literature. Indeed, leaving *V*_0_ free to vary during the fitting inevitably leads to unrealistic results. The EOS curve obtained by fitting all our data across the investigated pressure interval is plotted in Figure 8.

**Table 1:** EOS Parameters Determined by the Mathematical Fit of Experimental and Theoretical Data on As^a^

phase [pressure interval]	*B*_0_ (GPa)	*B*_0_^′^	*V*_0_^atom^ (Å^3^)	EOS type
This study				
A7 [0–26.5 GPa]	41.2 ± 1.4	6.2 ± 0.4	21.520 ± 0.008	BM3
p-sc [26.5–31.8 GPa]	63.9 ± 6.3	3.0 ± 0.5	21.528	BM3
p-sc [26.5–31.8 GPa]	109.0 ± 7.5	1.8 ± 0.4	20.10 ^21^	BM3
p-sc [26.5–31.8 GPa]	55.2 ± 6.0	3.2 ± 0.5	21.96 ^1^	BM3
A7/p-sc [0–31.8 GPa]	42.4 ± 1.2	5.7 ± 0.3	21.518 ± 0.008	BM3
p-sc/sc [26.5–37.8]	40.7 ± 7.1	5.9 ± 1.3	21.528	BM3
p-sc/sc [26.5–37.8]	79.9 ± 8.7	4.0 ± 0.8	20.10 ^21^	BM3
p-sc/sc [26.5–37.8]	33.3 ± 6.7	6.5 ± 1.6	21.96 ^1^	BM3
A7/p-sc/sc [0–37.8]	42.6 ± 1.1	5.7 ± 0.2	21.518 ± 0.008	BM3
Literature (experiments)				
A7 [0–31.4 GPa][Bibr ref5]	55.6	4.4	21.53	M
A7 [0–25 GPa][Bibr ref4]	58 ± 4	3.3 ± 0.4	21.53	BM3
sc [23.8–44.5 GPa][Bibr ref1]	38.3 ± 0.5	5.36 ± 0.09	21.96 ± 0.05	BM
A7 to sc [0–48.0 GPa][Bibr ref8]	53.8 ± 0.2	4.0 ± 0.2	21.522 ± 0.002	BM3
A7 to bcc [0–122][Bibr ref6]	56 ± 3	3.7 ± 0.2	-	BM3
Literature (theory)				
A7 (DFT-GGA-PBE)^21^	36.9 ± 1.6	6.1 ± 0.7	22.22 ± 0.05	BM3
A7 (DFT-GGA-PBE)^21^	36.9 ± 1.6	6.25 ± 0.55	22.22 ± 0.05	V
sc (DFT-GGA-PBE)^21^	78.99 ± 0.03	4.318 ± 0.004	20.10	BM3
sc (DFT-GGA-PBE)^21^	78.51 ± 0.16	4.55 ± 0.02	20.10	V

a B_0_, *B*_0_^′^, and *V*_0_^atom^, respectively, represent the zero pressure bulk modulus, its first derivative, and the atomic volume. The EOS type is indicated as M = Murnaghan, BM3 = third-order Birch-Murnaghan, and V = Vinet. The EOS parameters were derived by the orthogonal distance regression (ODR) method, taking into account experimental uncertainties on volume and pressure (Table SI-1). In the p-sc and p-sc/sc pressure ranges, *V*_0_^atom^ was fixed to our ambient pressure volume of A7 As–I (21.528(5) Å^3^) and to the zero pressure volume of sc As–II calculated by Silas et al. (20.10 Å^3^, DFT-GGA-PBE)^21^ and experimentally determined by Akahama et al. from high-pressure data (21.96 Å^3^).^1^

Finally, relevant insights emerge from our data by considering that interlayer interactions involving the electron LPs apparently begin to strengthen with density in the stability field of the A7 structure, where the interlayer distances reduce more than the intralayer ones, well before the onset of the p-sc structure (Figure 4). This is clearly revealed by the smooth and sluggish change in the slope of the pressure dependence of α, *u*, *a*, *d*_1_, and *d*_2_ at ∼8–10 GPa (Figure 3). Interestingly, the occurrence of subtle structural modifications in the interatomic distances between 10 and 22 GPa was suggested also by Beister et al., based on the flattening of the room *T* Raman phonon frequency ratio *R* = ν­(*E*_*g*_)/ν­(*A*_1*g*_) in this pressure interval and possibly related to the decrease of the resistivity and the onset of superconductivity at low temperature.^4^ In this regard, despite not correlating their findings to the Raman data by Beister et al., Silas et al. observed the opening of an electron pocket at 8–10 GPa at the *T* point of the first Brillouin zone (BZ), while calculating the pressure dependence of the Fermi surface of As across the A7 to sc phase transition.^22^ In agreement with their previous study,^21^ they found the phase transition to take place at 27 GPa, when the hole pockets appearing at the *W* point of the first BZ connect on opposite sides with two neighboring electron pockets, one of which corresponds to the electron pocket opened at the *T* point at 10 GPa.^22^ In this respect, a recent DFT theoretical study has reported superconductivity to originate from broken-symmetry atomic configurations, in which the electron–phonon interaction creates resistance-free straight one-dimensional tunnels (SODTs) for charge density of electrons and/or holes,^69^ that are related to the Cooper pairs in the Bardeen–Cooper–Schrieffer theory.^70^ In the case of As, these tunnels are not present at ambient conditions, but their formation is seemingly induced by pressure, in agreement with the calculations by Silas et al.^22^

Moreover, according to the experiments by Chen et al.^13^ and to the calculations by Chan et al.,^14^ the pressure dependence of the superconducting *T*_c_ of As exhibits an asymmetrically shaped maximum at ∼32 GPa, which appears to be in excellent agreement with the observation of the p-sc structure in the 26.5–31.8 GPa pressure range and with the attainment of the sc structure at 31.8 GPa. While these phenomena have been traditionally interpreted based on the A7 and sc structures, with most recent experimental studies providing low sampling resolution in this pressure range and contrasting results at higher pressure,^13,32,34,49,50,71–73^ the discovery of the intermediate p-sc structure in the phase diagram of As at room *T* may suggest the existence of a different underlying crystal structure and help shed light on the controversial pressure dependence of the superconducting *T*_c_ in As.

## Conclusions

The results of this study provide in-depth structural and chemical insights into the long-debated prototypical phase transition from layered A7 As–I to nonlayered sc As–II. Taking advantage of single-crystal structure determination by synchrotron XRD in DAC, we report the pressure dependence of the rhombohedral lattice parameters and interatomic distances of As during room-*T* compression in He.

Our data unveil a two-step pressure-induced mechanism driving the interlayer bond formation process across the A7 to sc phase transition. The first step takes place at 26.5 GPa, when the attainment of α = 60° marks a rhombohedral to simple cubic lattice transition, whereas the second step takes place at 31.8 GPa, when *u* finally reaches the 0.250 value, indicating that atoms have finally moved to the expected sc sites. This experimental evidence demonstrates that a pseudo simple-cubic structure exists in the phase diagram of As between 26.5 and 31.8, analogous to that reported in P.^35,36^

Furthermore, the pressure dependence of the first and second neighbor interatomic distances reveals the occurrence of a *trans-*influence effect in the pressure range of the p-sc structure, underlying the interlayer bond formation mechanism and leading to the increase in the coordination number of As from 3 to 6. From a chemical point of view, the discovery of the p-sc structure in As, bridging the transition from A7 As–I to sc As–II, highlights the persistence of *s-p* orbital mixing, which favors LP electron density localization and trigonal pyramidal over octahedral atomic coordination, while preventing the attainment of the full symmetry of the simple cubic structure by the hindered atomic occupation of the sc sites.

While solving a long-standing structural debate on As, this study provides experimental evidence for the pressure-driven interlayer bonding mechanism underlying the increase of the density of states at the Fermi level across the associated semimetal-to-metal transition.^21,22,27,55^ Additional insights concern the onset of superconductivity and the pressure dependence of *T*_c_ in As. The results are relevant to chemical bond theory and to the high-pressure synthesis of innovative As-based materials of energetic and technological interest.

## Supplementary Material



## Notes

Experimental details, data, and discussion, including images and methods (PDF)
