# Supplementary material for: Pressure-Induced Interlayer Bonding in Arsenic Revealed by Single-Crystal Synchrotron X‑ray Diffraction in a Diamond Anvil Cell
Source: J Am Chem Soc. 2026 Sep 23;148(39):41999–2011. doi: 10.1021/jacs.6c09191 (PMC13651519; doi:10.1021/jacs.6c09191)
Supplement: Supplementary file 1 [file ja6c09191_si_001.pdf]

Supporting Information
Pressure-Induced Interlayer Bonding in Arsenic Revealed by Single-Crystal Synchrotron X-ray Diffraction in a Diamond Anvil Cell

Matteo Ceppatelli,[*,†,‡] Marta Morana,[¶] Enrico Berretti,[†] Kamil Dziubek,[§] Konstantin Glazyrin,[‖] Demetrio Scelta,[†,‡] Alessandro Lavacchi,[†] Roberto Bini,[‡,⊥,†] Maurizio Peruzzini,[†] and Manuel Serrano-Ruiz[†]

†*ICCOM-CNR, Institute of Chemistry of OrganoMetallic Compounds, National Research Council of Italy, Via Madonna del Piano 10, I-50019 Sesto Fiorentino, Firenze, Italy*

‡*LENS, European Laboratory for Non-linear Spectroscopy, Via N. Carrara 1, I-50019 Sesto Fiorentino, Firenze, Italy*

¶*Dipartimento di Scienze della Terra, Università degli Studi di Firenze, Via La Pira 4, I-50121 Firenze (FI), Italy.*

§*Department of Mineralogy and Crystallography, Universität Wien, Josef-Holaubek-Platz 2 (UZA II), 1090 Wien, Austria*

‖*Deutsches Elektronen-Synchrotron DESY, Notkestr. 85, 22607 Hamburg, Germany*

⊥*Dipartimento di Chimica "Ugo Schiff"dell'Università degli Studi di Firenze, Via della Lastruccia 3, I-50019 Sesto Fiorentino, Firenze, Italy*

E-mail: matteo.ceppatelli@iccom.cnr.it,ceppa@lens.unifi.it

Contents

SI-1 Scientific background and relevant literature **3**

SI-2 Ambient pressure As structures **7**

SI-3 Purification and synthesis of single-crystal arsenic **8**

SI-4 Carving and extraction of small As single crystals by Focused Ion Beam processing of a large As single crystal **9**

SI-5 DAC loading and pressure generation **11**

SI-6 High-pressure single-crystal X-ray diffraction: data acquisition and analysis **13**

SI-7 The A7 to p-sc and p-sc to sc phase transitions **23**

SI-8 Pressure-dependent orbital contributions and electrostatic interactions **25**

Acknowledgements **27**

Author contributions **27**

Competing interests **28**

Supplementary References **28**

SI-1 Scientific background and relevant literature

Experimentally, two studies have set the benchmark for any theoretical investigations so far.

Kikegawa and Iwasaki[1] performed an energy-dispersive powder synchrotron XRD study of the A7 to sc phase transition of polycrystalline As in DAC using fluid paraffin as PTM, reporting the phase transition to occur at ∼32 GPa and to have first-order character. Specifically, they observed a discontinuity between two consecutive pressure points at 31.4 and 36.6 GPa, with no intermediate data points, in the pressure dependence of the c/a hexagonal lattice parameter ratio and of the atomic volume. From the intensity ratio of the 003 and 006 reflections, they estimated u=0.237±0.002 Å at 31.4 GPa, thus concluding As to be still in the A7 structure at this pressure, and observed the characteristic pattern of the sc structure at 36.6 GPa, which they followed up to 44.7 GPa. Although in the abstract of the article Kikegawa and Iwasaki located the phase transition at ∼32 GPa, we were not able to find any evidence supporting the precise identification of this pressure from the presented data, as also noticed by other studies,[2–4] in which the 34 GPa value at the middle of the 31.4-36.6 GPa pressure interval was selected as the transition pressure emerging from the data published by Kikegawa and Iwasaki.[1]

A few years later Beister *et al.*[2] investigated the A7 to sc phase transformation in As by angle-dispersive powder synchrotron XRD and single-crystal Raman spectroscopy. They acquired powder XRD patterns of As in DAC during room T compression up to 28 GPa, using methanol-ethanol (4:1) as PTM, and identified the phase transition to occur at 25 GPa by the merging of the 110 and 104 reflections of the A7 structure (hexagonal setting indexing) into the 110 reflection of the sc one (cubic setting indexing). In their study no discontinuity in the pressure dependence of the relative volume change $\left(\frac{\Delta V}{V_0}\text{, where } \Delta V = V - V_0\right)$ was observed in the investigated pressure range, whereas a slight discontinuity emerges at ∼25 GPa from the pressure dependence of the relative variations of the hexagonal lattice parameters a and c $\left(\frac{\Delta x}{x_0}\text{, where } \Delta x = x - x_0 \text{ and } x = a, c\right)$. Particularly, while $\frac{\Delta a}{a_0}$ exhibits

a slight positive increase before further continuing to decrease with pressure, $\frac{\Delta c}{c_0}$ exhibits a marked negative drop. They also observed a sharp discontinuity in the pressure dependence of α, plotted as $\alpha\left(\frac{\Delta V}{V_0}\right)$, reporting α to reach the 60° characteristic value of the sc structure at 25 GPa. Beister *et al.* could not experimentally determine the atomic position parameter u and the first (d_1) and second (d_2) neighbor distances from their powder XRD data. However, using an empirical $u(\alpha)$ relation, suggesting a smooth continuous pressure dependence of u, they estimated the pressure dependence of the first and second neighbor distances, showing an abrupt merging of d_1 and d_2 into a single value at 25 GPa and hence indicating the A7 to sc phase transition to occur sharply at this pressure. Overall, considering also their Raman data, they concluded the A7 to sc phase transition to be a first or nearly-first order transition.

Although likely representing the state-of-the-art of the experimental technique at the time of publishing and providing precious information and insights for future studies, the XRD studies by Kikegawa and Iwasaki[1] and by Beister *et al.*,[2] are however affected by critical limitations.

Both studies performed powder synchrotron XRD on As using a non-hydrostatic pressure transmitting medium: liquid paraffin in the case of Kikegawa and Iwasaki[1] and a methanol-ethanol (4:1) mixture in the case of Beister *et al.*[2] As an additional drawback, methanol and ethanol are highly prone to water contamination and not chemically inert with respect to As.[5]

Due to characteristic heavy texturing related to preferential orientations, which typically affects the intensity of the diffraction rings, Rietveld refinement of the layered A7 As-I structure at high-pressure is extremely difficult. Experimentally, this occurrence prevents a full structural determination and essentially allows only the extraction of the unit cell and lattice parameters, with no direct information on the atomic positions, whose precise determination is instead critical in this case. This is clearly stated by Beister *et al.*, when discussing the limitation of their empirical $u(\alpha)$ relation used to derive the pressure dependence of u and of d_1 and d_2, actually recommending single-crystal high-pressure studies.[2]

Furthermore, on one hand, the low resolution of the pressure sampling performed by Kikegawa and Iwasaki[1] was not sufficient to solve the subtle structural modifications underlying the phase transition, with only four pressure points between 25.0 and 36.6 GPa and no data points between 31.4 and 36.6 GPa, where they reported the phase transition to occur based on the discontinuity in the c/a ratio and atomic volume. It is also worth to mention that energy-dispersive XRD is affected by several drawbacks compared to angle dispersive XRD. The lower resolution of diffraction peaks limits the precision in the determination of unit-cell parameters, making the detection of subtle structural changes harder. Additionally, as the reliability of results is highly dependent on accurate calibration of the 2θ scattering angle and on the detector energy, small errors in the detector position can result in larger errors in spacing compared to angle-dispersive XRD.

On the other hand, an insufficient pressure range was covered by Beister and coauthors. This was likely related to the fact that the merging of the two A7 reflections into the single peak of the sc one was used as transition criterion. It was demonstrated in the case of P, that, when this happens for α=60°, weak reflections not expected in the sc pattern remain visible until the value of the atomic position parameter u remains lower than the 0.250 limit value characteristic of the sc structure.[6,7] Looking only at the merging of the 110 and 104 reflections has thus possibly led Beister *et al.* to underestimate the transition pressure to the sc structure.

On the theoretical side, various computational studies have modeled the A7 to sc phase transition in As. However, the small energy difference between the two structures and the rapid change in the density of states (DOS) across the Fermi level, requiring high levels of convergence, have represented a computational challenge for long time, leading to calculated transition pressures scattered from 19 to 36 GPa.[3,4,8–18] Silas *et al.* performed DFT calculations on the crystal structures of As under compression up to 200 GPa, using adequate k-sampling of the Brillouin zone and provided a description of the A7 to sc phase transition in terms of α, u, d_1 and d_2, locating the transition pressure at 21±1 (LDA), 28±1 (GGA-PBE)

or 29±1 GPa (GGA-PW91), depending on the adopted approximations. A similar result was later obtained by Chan *et al.*,[17] who located the A7-sc phase transition between 20 and 25 GPa. Interestingly, focusing their calculations on the electron-phonon coupling constant λ, Chan *et al.* found the superconducting transition temperature T_c to increase with pressure in the A7 structure, reaching a maximum at the A7 to sc phase transition and immediately starting to decrease at higher pressure.

SI-2 Ambient pressure As structures

Besides As-I, two other crystalline allotropes of As are known at ambient pressure. Yellow arsenic is an unstable solid made of As_4 molecules, whose crystal structure has never been determined,[19] while black arsenic, rarely found in natural minerals (arsenolamprite),[20,21] exhibits a layered crystal structure isostructural to orthorhombic black phosphorus ($Cmce$, Z=8) and is therefore named black arsenic. Black As is metastable at ambient pressure and rapidly converts into gray As (As-I) under moderate compression (0.7-5.4 GPa).[22–24]

A crystal structure named pararsenolamprite ($Pnm2_1$), consisting of the ordered stacking of layers made alternating rods of gray and black As, has been also reported.[25]

SI-3 Purification and synthesis of single-crystal arsenic

Pure single-crystal gray arsenic was obtained by recrystallizing poly-crystalline gray arsenic using a modified version of the Jeavons technique[26,27] A lump of polycrystalline gray As was purchased from Alfa Aesar (99,999%, 150-200 mg) and placed into a quartz tube (length 10 cm, outer dimeter 13 mm, inner diameter 10 mm) under N_2 atmosphere. The quartz tube was sealed under vacuum and then heated to 730 ℃ at 2 ℃/min rate. The sample was held at this temperature for 10 h, then slowly cooled at 0.06 ℃/min rate, finally obtaining one large high-purity single crystals of gray-As of about 4 x 3 x 3 mm^3 size (Figure 2), whose purity and quality were checked and confirmed by single crystal X-ray diffraction at CRIST (Centro di Servizi di Cristallografia Strutturale) in Sesto Fiorentino.

SI-4 Carving and extraction of small As single crystals by Focused Ion Beam processing of a large As single crystal

Small trigonal prisms (46 x 20 x 8 μm) of As single crystal, suitable for high-pressure single-crystal structure determination in DAC, were extracted from a large As monocrystal using the fabrication capabilities of a Tescan Gaia 3 FIB/SEM microscope at CNR-CEME.

The dimensions of the small single-crystal trigonal prisms of As were selected to fit inside the sample chamber without any contact with the walls of the gasket hole, Au and ruby, and to avoid the bridging of the sample during the compression (Figure 2).

Firstly, an horizontal pillar-like structure was produced on the surface of the monocrystal using a Ga Ion beam with an energy of 30 keV (Figure SI-1a). The end of the pillar was then soldered using Pt organometal gas to a nano-manipulator with a needle tip of 1 μm. The top-end of the pillar was then cut by the same Ga ion beam to obtain a standalone sample of about 10 μm depth (Figure SI-1b). The needle with the sample was consequently positioned on small box-shaped holes, previously carved onto an [111] Si wafer surface (from University wafers, certified flatness) by the same Ga Ion beam (Figure SI-1d and SI-1e). Finally, the sample was detached inside the hole in order to preserve it for transportation (Figure SI-1f), using another portion of Si wafer as a box lid. EDX analysis performed after the final deposition of the sample highlighted no Ga or Pt contamination. The small As single crystals were stored under inert N_2 atmosphere to prevented air exposure. The sample was then subject to a 5 keV Ion beam irradiation in order to get rid of both eventual amorphization, and Ga contamination (Figure SI-1c).

The crystal quality of the sample after synthesis and FIB processing was checked at ambient conditions by XRD using a Bruker D8 Venture single-crystal diffractometer at the CRIST facility in Sesto Fiorentino.

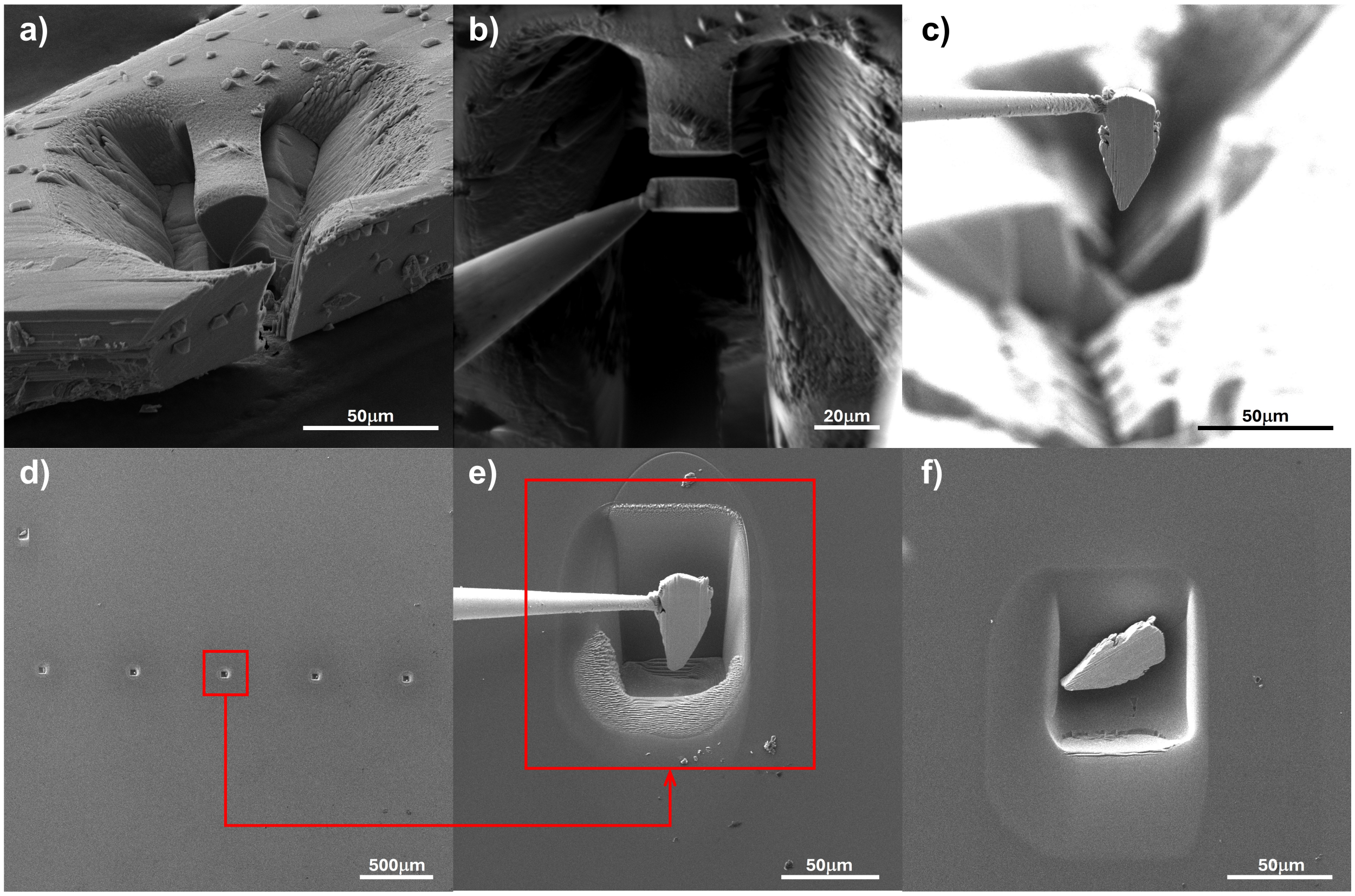

Figure SI-1: Microscope images (see text for details). a) Horizontal pillar-like structure excavated on the surface of the As monocrystal using a Ga Ion beam with an energy of 30 keV. b) End of the pillar of single-crystal As soldered by a Pt organometal gas to the needle tip (1 μm) of a nano-manipulator and cut by the Ga ion beam. c) Small As single-crystal attached to the nano-manipulator tip after 5 keV ion beam irradiation to get rid of both eventual amorphization, and Ga contamination. d)Box-shaped holes carved onto a [111] Si wafer surface (from University wafers, certified flatness) by the Ga ion beam to host the small As single crystal during transportation. e) Small As single-crystal attached to the nano-manipulator tip, before positioning into the box-holes. f) Small As single-crystal deposited inside a box-hole.

SI-5 DAC loading and pressure generation

Pressure was generated using membrane Diamond Anvil Cells (DACs) equipped with Ia type standard cut 16-sided anvils having 300 μm culets. Re gaskets 200 µm thick were indented to 45 μm thickness and laser-drilled to obtain a 150 μm sample chamber.

The manipulation of the small single crystals by metallic tips, to place them inside the DAC, inevitably introduces defects and strain deteriorating the crystal structure, as confirmed by acquisition of XRD patterns, showing the elongation of the single crystal spot reflections.

This occurrence was avoided by preparing micrometric tips of paper fibers, which enable firm grip of the small As single crystal without application of detrimental force, thus ensuring the preservation of the crystal quality during its delicate positioning at the center of the gasket hole.

Au and a ruby chip for pressure measurement were placed inside the gasket hole, far enough to avoid contact with the As single crystal. The pressure values listed in Table SI-1 were obtained by the ruby photoluminescence method, using the ruby pressure calibration by Shen *et al.*[28] According to this calibration, the measured pressure values are affected by a 2.5% uncertainty in the investigated pressure range. High-purity He (5.0 grade) was then loaded into the DAC by the gas-loading technique. The gas-loading system was preliminarily purged and flushed with an overpressure of He (1.5-2.0 bar) for 30 s (Figure SI-2).

The photomicrographs of the sample, acquired on the two sides of the DAC, respectively before compression and after decompression, likely indicate the absence of bridging during the experiment, as suggested by the shiny appearance of the side of the sample lying on one diamond and by the dark aspects of the other side. The last occurrence indicates that this side of the sample was not directly squeezed by the other diamond and did not come in contact with it.

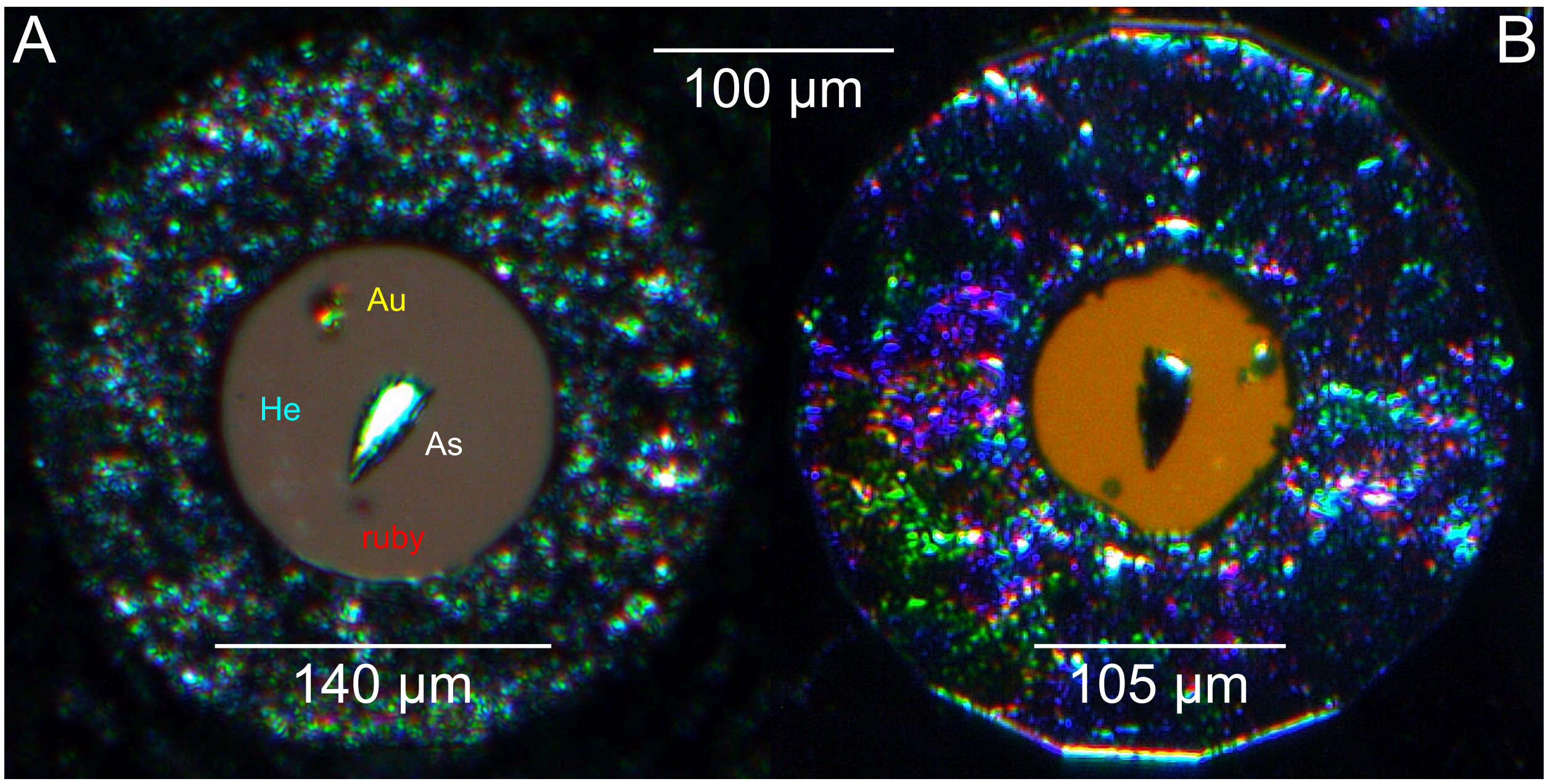

Figure SI-2: A. Photomicrograph of the small As single crystal inside the DAC at 0.1 GPa and room T after gas-loading He and before starting the compression. Labels indicate the Au and ruby pressure sensors. The transparent area corresponds to fluid He. The image was acquired from the side of the cell where the As crystal was placed in contact with the diamond surface, as attested by the shiny reflecting surface of the crystal. B. Photomicrograph of the recovered As single crystal inside the DAC at ambient conditions after room T compression up to 37.8 GPa and further decompression. The image was acquired immediately after releasing pressure at the end of the experiment from the opposite side of the cell compared to A.

SI-6 High-pressure single-crystal X-ray diffraction: data acquisition and analysis

High-pressure single-crystal synchrotron X-ray diffraction patterns were acquired at the P02.2 beamline (PETRA-III, DESY).[29] The monochromatic X-ray beam with 0.2897 Å wavelength was focused to a 8x3 μm^2 beam spot size and diffraction patterns were acquired using a Perkin Elmer XRD1621 detector (2048x2048 pixels, 200x200 μm^2).

Enstatite was used for the calibration of the single-crystal instrumental model, resulting in a sample to detector distance of 401.58 mm. Diffraction intensities were acquired in an ω-oscillation scan mode ($\pm 27°$ range, 0.5° frame width, 0.50 s exposure per frame) using Pt filters of variable thickness ranging between 25 and 225 μm, and the diffraction images were processed using a CrysAlisPro suite.[30] The structure was solved by direct methods using SHELXT[31] and refined by full-matrix least-squares calculations using SHELXL program suite.[32] No evidence of twinning, satellite reflections or diffuse scattering was found at the visual inspection of the area detector images. First (d_1) and second (d_2) neighbor distances and related uncertainties were checked by the *Crystal Palace* software for parametric crystal structure analysis.[33] Results of the single-crystal XRD data analysis are listed in Table SI-1.

Panoramic oscillation images were acquired to check the sample during the compression. In this case the setup was calibrated against CeO_2 powder standard and Dioptas[34] software used for integrating the 2D area images to 1D patterns. A detector image of XRD from single-crystal As in DAC after gas-loading He as PTM at 0.3 GPa and room T is shown in Figure SI-4, whereas XRD oscillation images acquired during compression are shown from Figure SI-5 to Figure SI-10.

P (GPa)	a_{hex} (Å)	c_{hex} (Å)	V_{hex} (Å^3)	V_{hex}^{atom} (Å^3)	a_{rho} (Å)	α_{rho} (degree)	V_{rho} (Å^3)	V_{rho}^{atom} (Å^3)	c/a	u	d_1 (Å)	d_2 (Å)
0	3.7599(2)	10.551(2)	129.17(3)	21.528(5)	4.1330(6)	54.112(9)	43.057(10)	21.528(5)	2.8062(2)	0.22738(5)	2.5207(6)	3.1163(9)
0.33(1)	3.7576(2)	10.470(3)	128.02(4)	21.337(7)	4.1093(9)	54.414(13)	42.670(13)	21.340(7)	2.7864(3)	0.22750(9)	2.5158(10)	3.1013(15)
1.38(3)	3.7502(3)	10.259(3)	124.95(5)	20.825(8)	4.0475(8)	55.197(14)	41.650(17)	20.825(8)	2.7356(3)	0.22885(9)	2.5131(10)	3.0469(14)
2.94(7)	3.7360(2)	10.058(4)	121.58(5)	20.263(8)	3.9866(11)	55.883(18)	40.527(17)	20.263(8)	2.6922(4)	0.23008(11)	2.5059(12)	2.9944(17)
4.60(12)	3.7222(2)	9.871(3)	118.44(3)	19.740(5)	3.9300(8)	56.532(14)	39.480(10)	19.740(5)	2.6519(3)	0.23129(15)	2.4992(16)	2.946(2)
6.42(16)	3.7078(3)	9.722(3)	115.74(4)	19.290(7)	3.8839(8)	57.023(15)	38.580(13)	19.290(7)	2.6220(3)	0.23244(15)	2.4936(16)	2.904(2)
8.3(2)	3.6922(2)	9.595(3)	113.28(4)	18.880(7)	3.8436(8)	57.410(14)	37.760(13)	18.880(7)	2.5987(3)	0.23325(15)	2.4853(13)	2.8693(18)
9.7(2)	3.6802(3)	9.502(3)	111.45(4)	18.575(7)	3.8140(8)	57.692(15)	37.150(13)	18.575(7)	2.5819(3)	0.23374(14)	2.4778(14)	2.8455(19)
10.8(3)	3.6738(3)	9.445(3)	110.40(4)	18.400(7)	3.7962(8)	57.878(15)	36.800(13)	18.400(7)	2.5709(3)	0.23460(13)	2.4791(13)	2.8244(17)
12.2(3)	3.6636(3)	9.376(3)	108.99(4)	18.165(7)	3.7738(8)	58.077(15)	36.330(13)	18.165(7)	2.5592(3)	0.23512(11)	2.4742(11)	2.8046(15)
13.5(3)	3.6545(3)	9.323(3)	107.83(4)	17.972(7)	3.7562(8)	58.216(15)	35.943(13)	17.972(7)	2.5511(3)	0.23559(12)	2.4705(13)	2.7881(17)
15.0(4)	3.6422(3)	9.250(4)	106.27(5)	17.712(8)	3.7321(11)	58.41(2)	35.423(17)	17.712(8)	2.5397(4)	0.23613(16)	2.4644(16)	2.767(2)
16.2(4)	3.6393(4)	9.216(4)	105.71(6)	17.618(10)	3.7218(11)	58.54(2)	35.24(2)	17.618(10)	2.5324(4)	0.2366(3)	2.465(3)	2.756(4)
17.6(4)	3.6302(4)	9.168(4)	104.63(5)	17.438(8)	3.7057(11)	58.66(2)	34.877(17)	17.438(8)	2.5255(4)	0.2372(4)	2.463(4)	2.739(5)
18.8(5)	3.6027(5)	9.075(5)	102.01(6)	17.002(10)	3.6711(14)	58.77(3)	34.00(2)	17.002(10)	2.5189(6)	0.2378(2)	2.448(2)	2.708(3)
20.1(5)	3.6025(10)	9.058(11)	101.80(13)	16.967(22)	3.6664(30)	58.85(6)	33.93(4)	16.97(2)	2.5144(12)	0.2385(2)	2.453(3)	2.698(3)
21.1(5)	3.6033(8)	9.041(9)	101.66(11)	16.943(18)	3.6620(25)	58.94(5)	33.89(4)	16.943(18)	2.5091(10)	0.2388(3)	2.455(3)	2.693(4)
22.0(6)	3.5817(8)	8.959(9)	99.54(11)	16.590(18)	3.632(3)	59.08(5)	33.18(4)	16.590(18)	2.5013(10)	0.2392(2)	2.442(2)	2.669(3)
23.0(6)	3.5909(15)	8.973(14)	100.20(18)	16.70(3)	3.639(4)	59.12(7)	33.40(6)	16.70(3)	2.4988(16)	0.2397(2)	2.453(3)	2.669(3)
23.6(6)	3.5766(13)	8.914(13)	98.76(16)	16.46(3)	3.618(4)	59.24(7)	32.92(5)	16.46(3)	2.4923(15)	0.23983(18)	2.442(2)	2.654(3)
24.3(6)	3.5791(7)	8.922(7)	98.97(9)	16.495(15)	3.6214(19)	59.23(4)	32.99(3)	16.495(15)	2.4928(8)	0.23995(19)	2.455(2)	2.655(3)
24.8(6)	3.5652(13)	8.865(15)	97.58(18)	16.26(3)	3.601(4)	59.34(8)	32.53(6)	16.26(3)	2.4865(17)	0.24050(19)	2.439(3)	2.636(3)
25.3(6)	3.5832(10)	8.914(10)	99.12(13)	16.52(2)	3.621(3)	59.32(5)	33.04(4)	16.52(2)	2.4877(12)	0.24054(17)	2.4524(19)	2.649(3)
26.3(7)	3.5694(17)	8.832(18)	97.5(2)	16.25(3)	3.594(5)	56.6(1)	32.50(7)	16.25(3)	2.474(2)	0.2414(3)	2.447(4)	2.624(4)
26.8(7)	3.5844(19)	8.763(19)	97.5(2)	16.25(3)	3.580(5	60.08(10)	32.50(7)	16.25(3)	2.445(2)	0.2409(5)	2.444(5)	2.628(6)
27.5(7)	3.5820(9)	8.735(9)	97.06(11)	16.177(18)	3.571(3)	60.20(5)	32.35(4)	16.177(18)	2.4386(11)	0.2420(4)	2.451(4)	2.612(5)
28.9(7)	3.5588(6)	8.695(6)	95.37(7)	15.895(12)	3.5527(16)	60.11(3)	31.79(2)	15.895(12)	2.4432(7)	0.2437(6)	2.453(6)	2.579(7)
29.6(7)	3.5552(11)	8.686(12)	95.08(14)	15.85(2)	3.549(3)	60.11(6)	31.69(5)	15.85(2)	2.4432(14)	0.2460(6)	2.472(7)	2.552(8)
30.3(8)	3.5562(7)	8.682(6)	95.09(8)	15.848(13)	3.5483(16)	60.15(3)	31.70(3)	15.848(13)	2.4414(7)	0.2468(5)	2.480(5)	2.544(6)
31.3(8)	3.5433(7)	8.664(8)	94.20(9)	15.700(15)	3.539(2)	60.08(4)	31.40(3)	15.700(15)	2.4452(9)	0.2490(5)	2.494(5)	2.514(6)
32.3(8)	3.5398(4)	8.662(2)	93.99(5)	15.665(8)	3.5374(6)	60.044(13)	31.330(17)	15.665(8)	2.4470(3)	0.2500(2)	2.50(2)	2.50(2)
33.4(8)	3.5306(15)	8.6544(16)	93.43(8)	15.572(13)	3.5323(7)	59.97(3)	31.14(3)	15.572(13)	2.4513(5)	0.2500(3)	2.496(5)	2.498(5)
34.4(9)	3.5256(15)	8.6432(18)	93.04(8)	15.507(16)	3.5276(7)	59.96(3)	31.01(3)	15.507(13)	2.4516(5)	0.2501(6)	2.494(6)	2.493(6)
35.2(9)	3.5268(11)	8.633(2)	93.00(6)	15.500(10)	3.5252(7)	60.03(2)	31.00(2)	15.500(10)	2.4478(4)	0.250(7)	2.492(4)	2.495(4)
36.8(9)	3.5145(6)	8.6111(16)	92.11(3)	15.352(5)	3.5151(5)	59.988(15)	30.70(10)	15.352(15)	2.4502(3)	0.2502(4)	2.487(4)	2.484(4)
37.8(9)	3.521(13)	8.614(3)	92.48(7)	15.413(12)	3.5181 (9)	60.05(3)	30.83(2)	15.413(12)	2.4465(5)	0.25060(15)	2.4948(17)	2.4826(17)

Table SI-1: Lattice parameters, unit cell volume (V) and atomic volume (V^{atom}) of the As crystal structure in hexagonal (hex) and rhombohedral (rho) settings as a function of pressure, obtained from single-crystal structure determination during room T compression of As in He. The c/a hexagonal lattice parameters ratio, the atomic position parameter u, the first (d_1) and second (d_2) neighbor interatomic distances, and related errors,[35] are also reported. Results are listed for hexagonal (a_{hex}, c_{hex}, V_{hex}, V_{hex}^{atom}) and rhombohedral (a_{rho}, α_{rho}, u, V_{rho}, V_{rho}^{atom}) settings. V_{rho} and V_{rho}^{atom} have been derived from V_{hex} and V_{hex}^{atom}, respectively, exploiting exact mathematical relations. The pressure values were obtained by the ruby photoluminescence method, using the pressure calibration by Shen *et al.*, which is affected by a 2.5% uncertainty in the investigated pressure interval.[28]

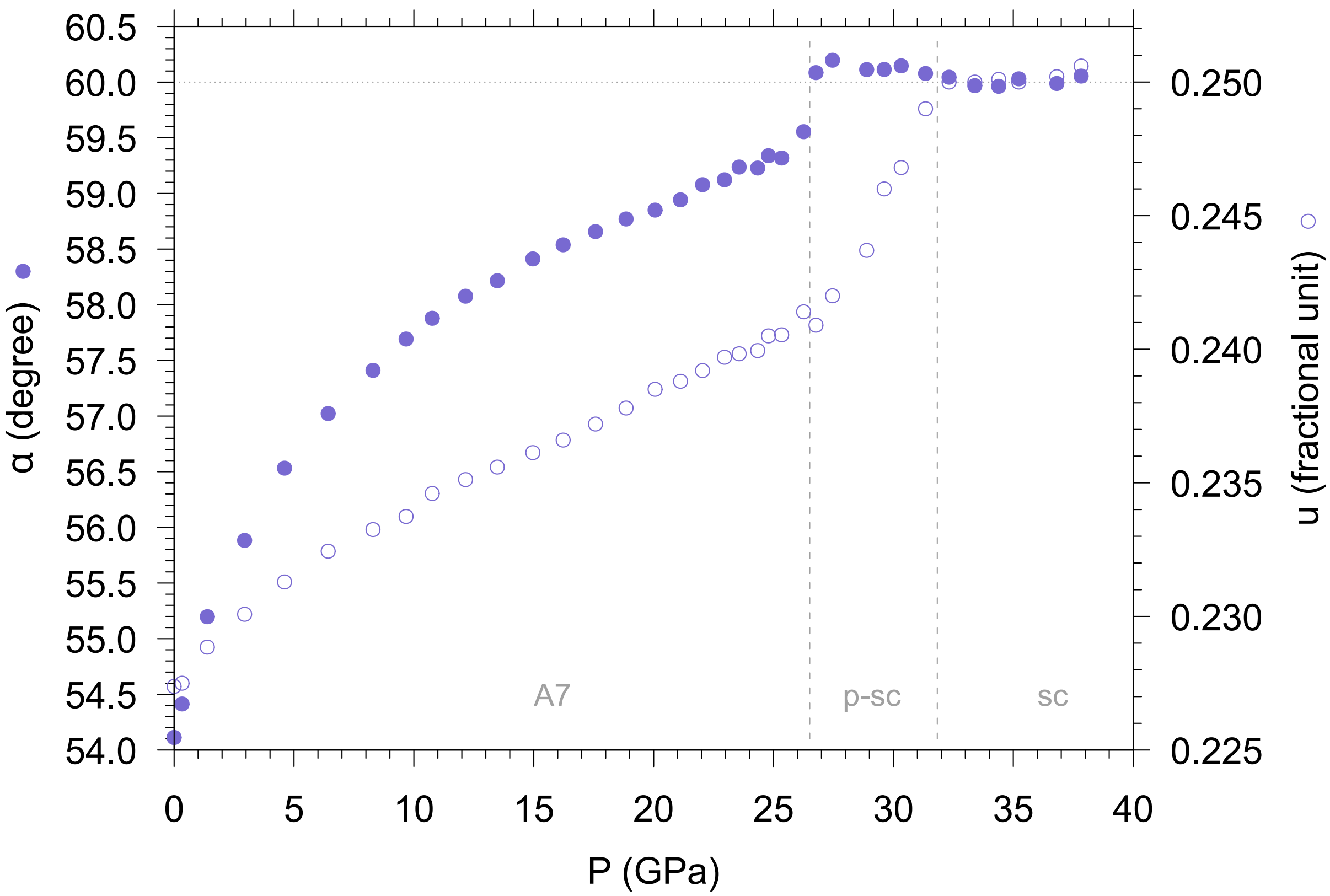

Figure SI-3: Pressure dependence of α (filled circles, left y-axis) and u (open circles, right y-axis). The gray vertical dashed lines at 26.5 and 31.8 GPa, respectively, mark the pressure values corresponding to the achievement of the α=60° and u=0.250 conditions, which are highlighted for both parameters by the horizontal dotted line. The pressure interval delimited by 26.5 and 31.8 GPa, where α=60°, while u<0.250, identifies the pressure range for the existence of the p-sc structure in As, between A7 As-I and sc As-II.

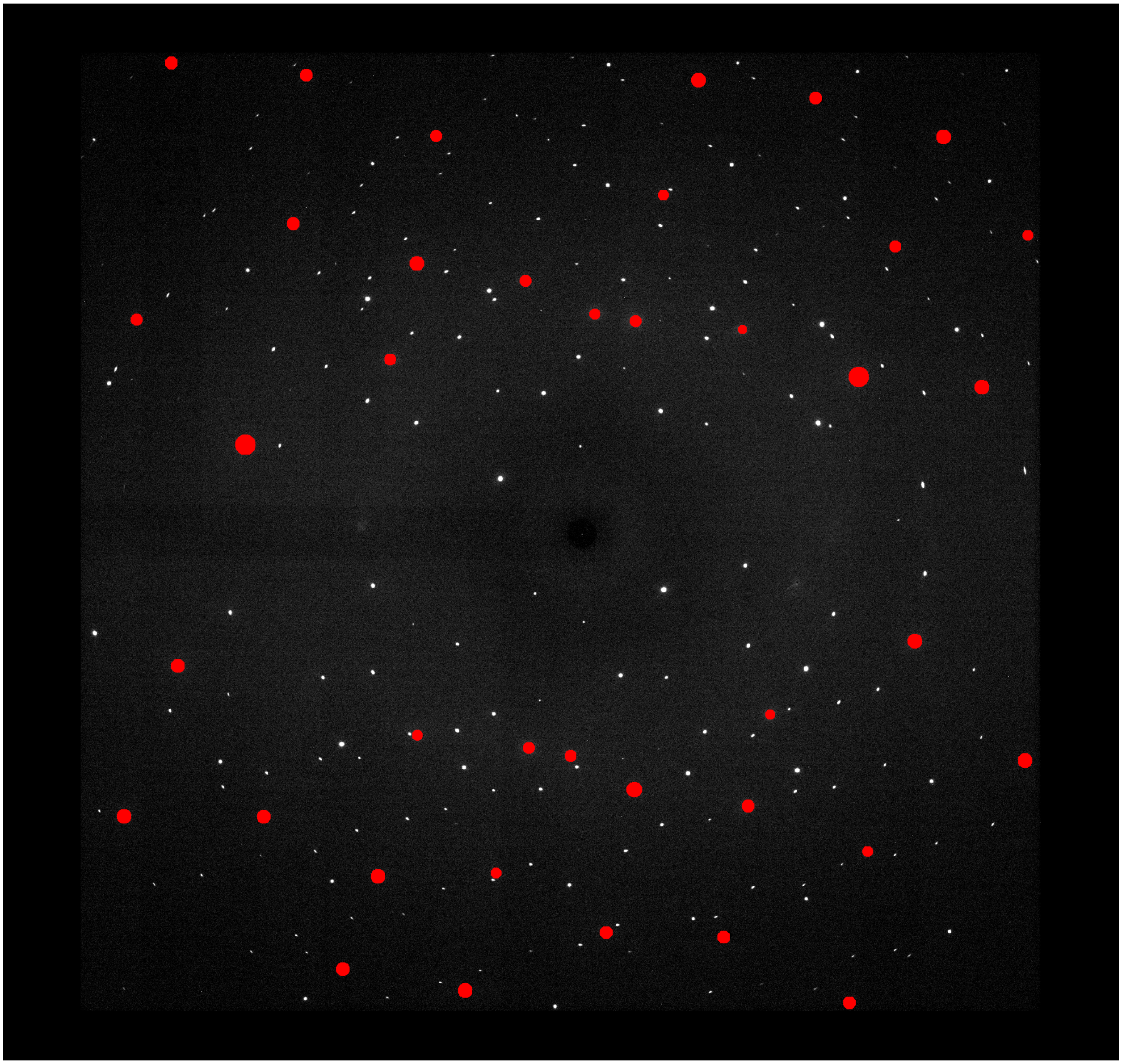

Figure SI-4: XRD oscillation image of single-crystal As in DAC at 0.3 GPa and room T after gas-laoding He, collected during a ±20 degree rotation. The red circles represent masked reflections from the diamond anvils.

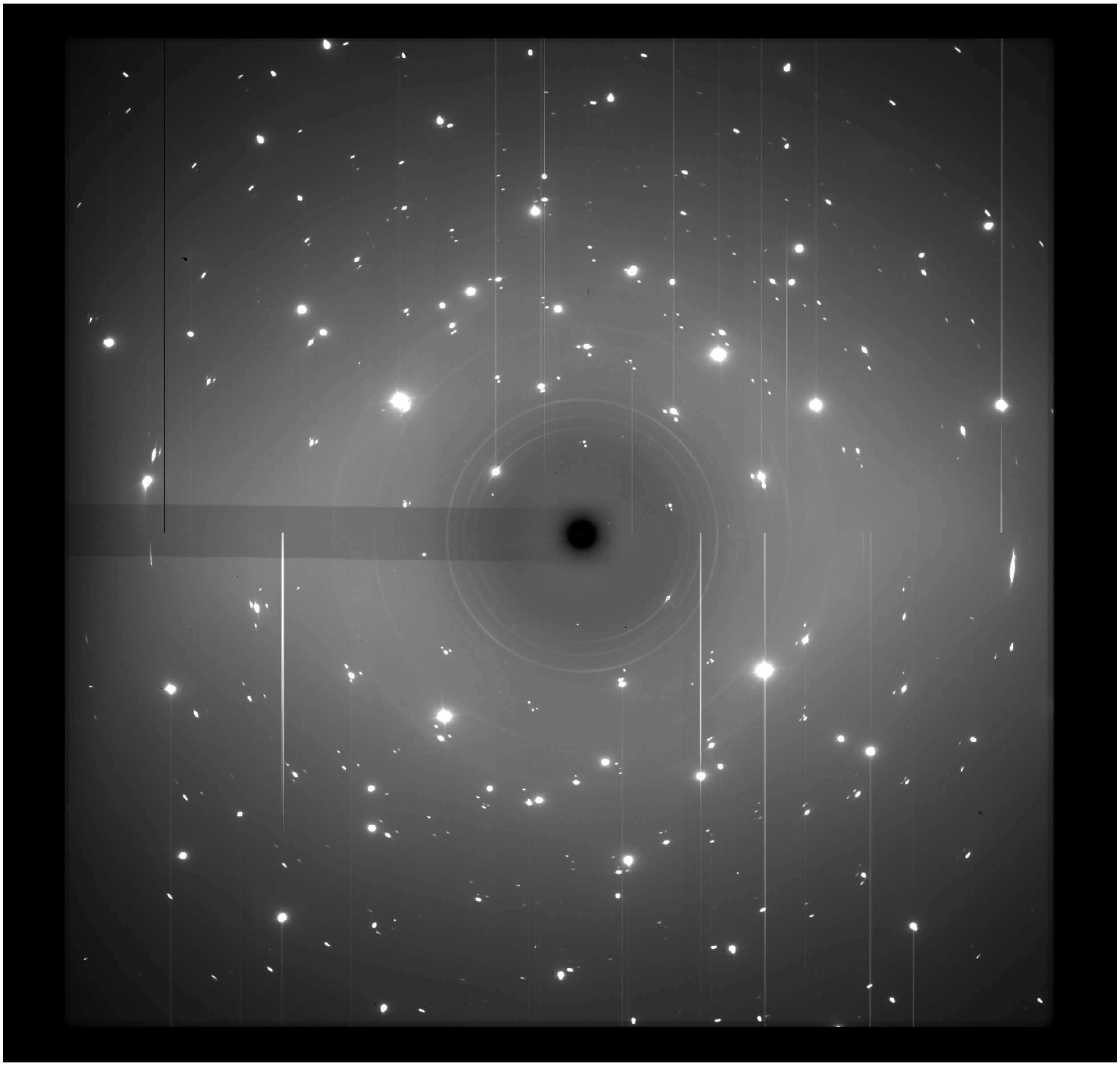

Figure SI-5: XRD oscillation image (±20°) of single-crystal As (A7) in He at 10.8 GPa and room T. No masking was applied.

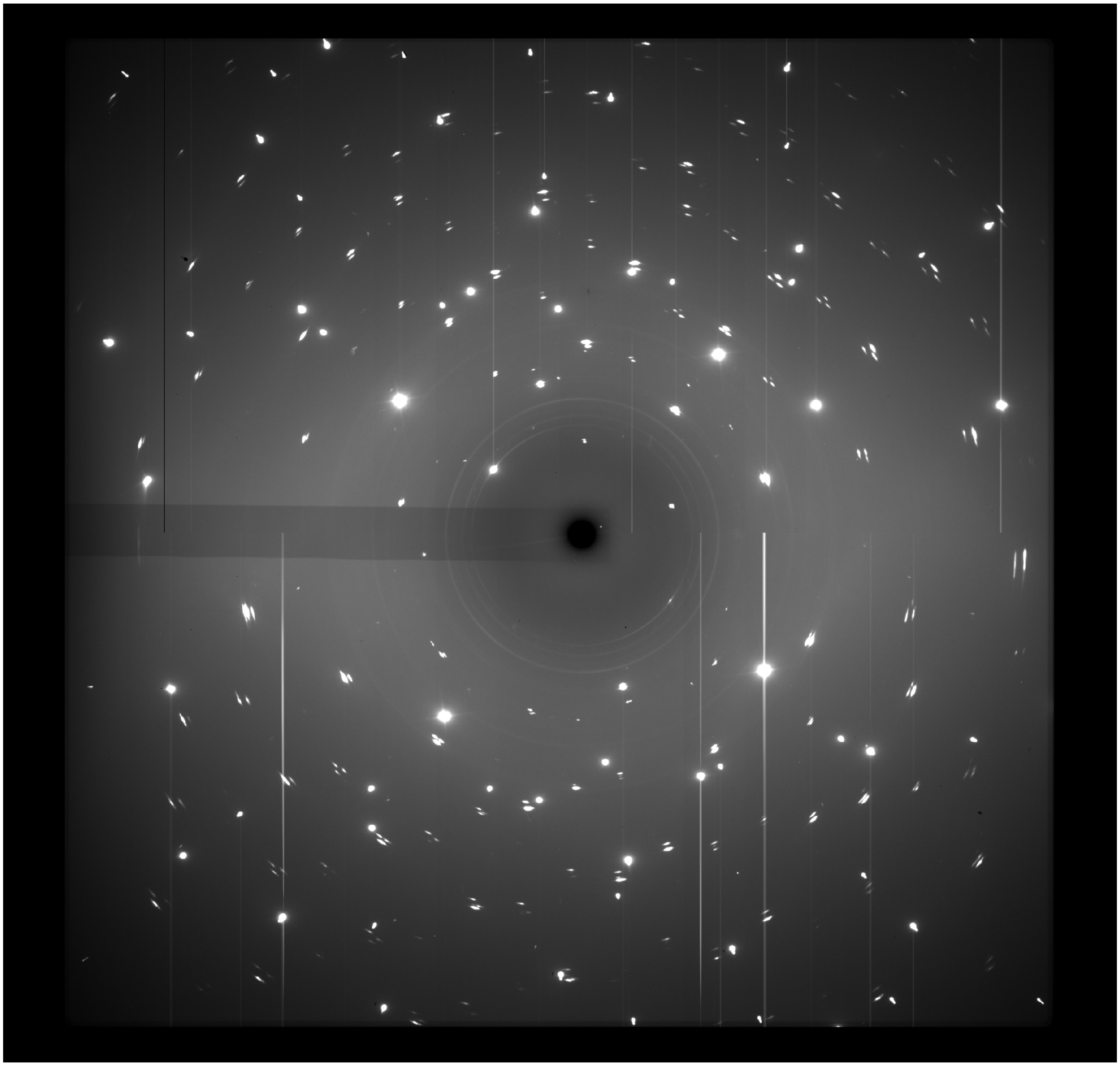

Figure SI-6: XRD oscillation image (±20°) of single-crystal As (A7) in He at 21.1 GPa and room T. No masking was applied.

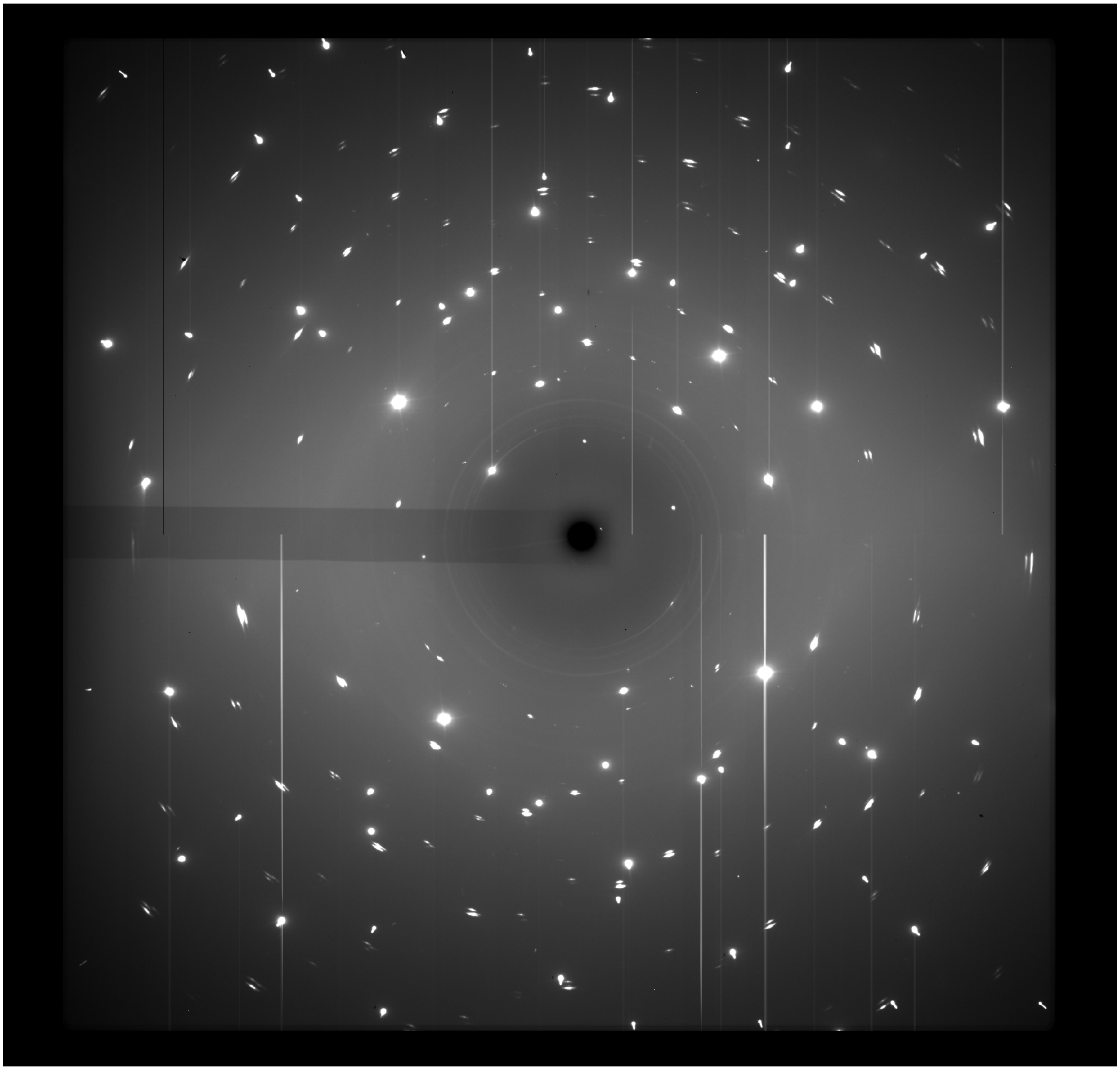

Figure SI-7: XRD oscillation image (±20°) of single-crystal As (A7) in He at 26.1 GPa and room T in the A7 structure. No masking was applied.

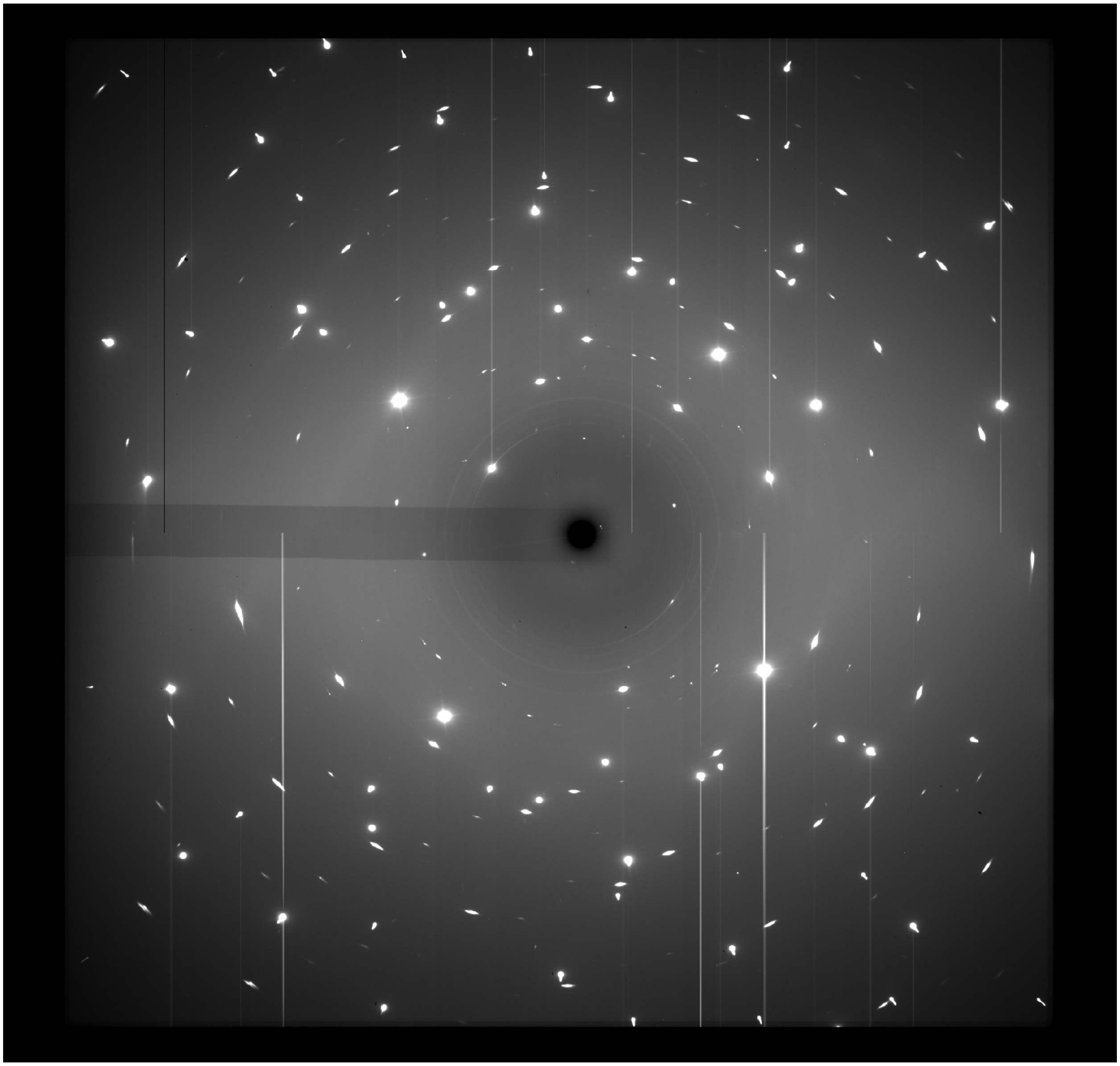

Figure SI-8: XRD oscillation image (±20°) of single-crystal As (p-sc) in He at 28.9 GPa and room T. No masking was applied.

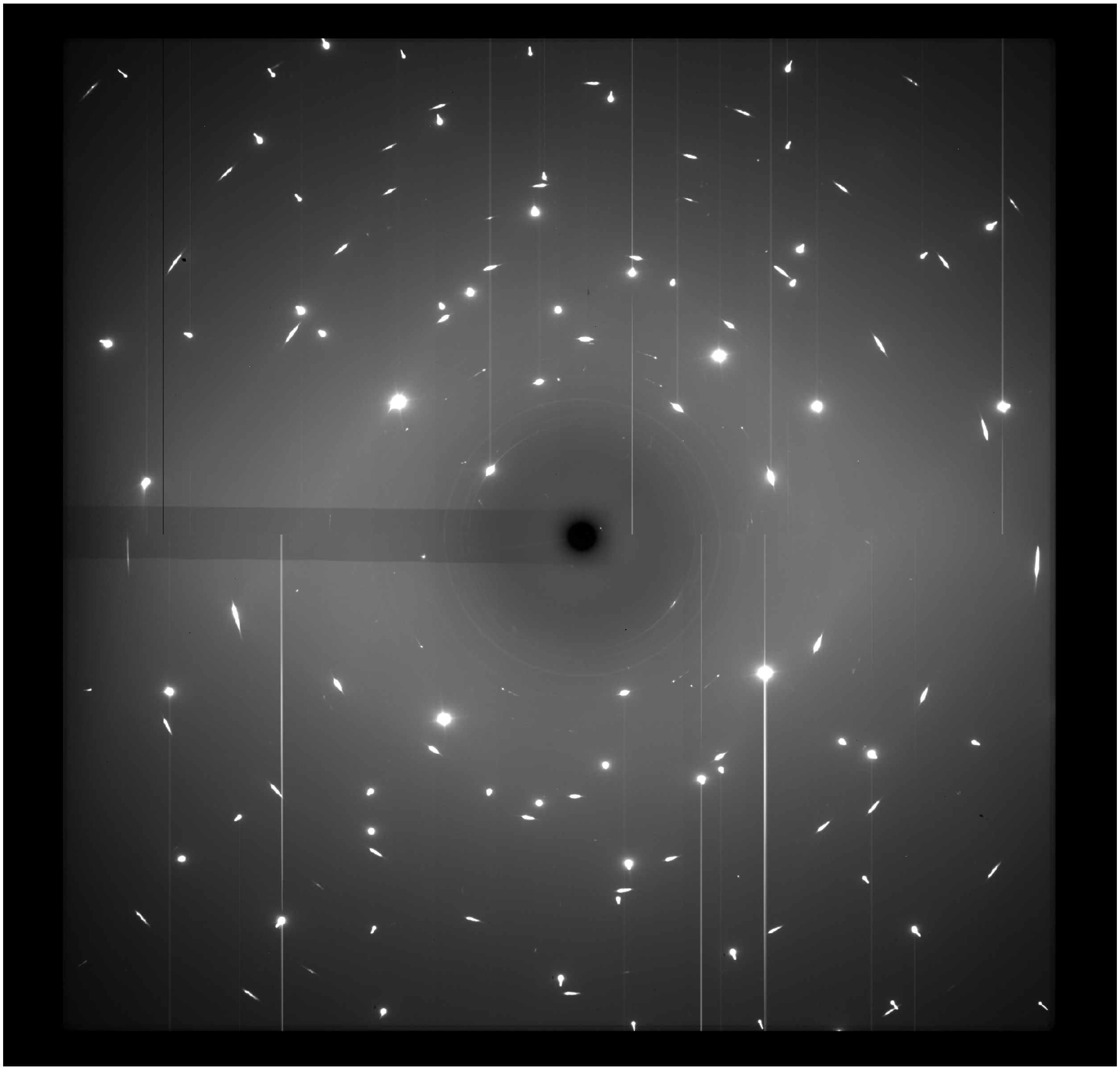

Figure SI-9: XRD oscillation image (±20°) of single-crystal As (sc) in He at 32.3 GPa and room T. No masking was applied.

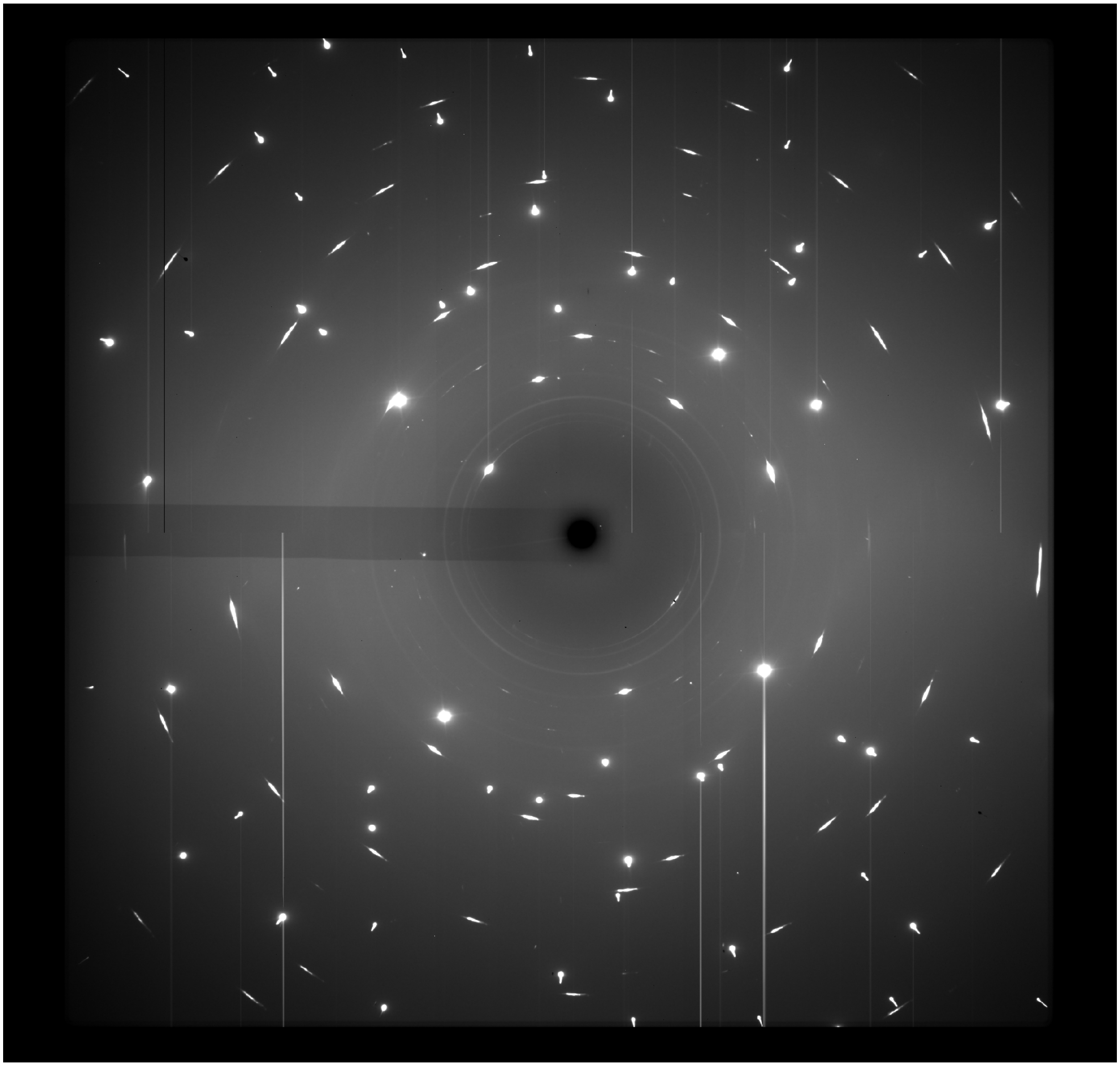

Figure SI-10: XRD oscillation image ($\pm 20^{\circ}$) of single-crystal As (p-sc) in He at 37.8 GPa and room T. No masking was applied.

SI-7 The A7 to p-sc and p-sc to sc phase transitions

The A7 structure can be ideally obtained from the sc one as the result of two structural modifications, respectively involving the elongation of the sc unit cell along one of the body cell diagonals and the displacement of the atoms along the same direction,[2,10,36] while the sc structure can be obtained from the A7 structure by the reverse process.

As crystal symmetry is concerned, the A7 to sc phase transition is associated to a symmetry increase, leading to the formation of octahedral atomic coordination with six equivalent bonds. Conversely, a symmetry reduction, also referred to in literature as symmetry breaking, is associated to the reverse process on releasing pressure, leading to the formation of three strong covalent bonds and three weaker secondary interactions with associated trigonal pyramidal atomic coordination and LP electron density localization.

The abrupt discontinuity in the pressure dependence of α at 26.5 GPa and the associated slope change in the pressure dependence of u suggest the A7 to p-sc transition, corresponding to the first step of the A7 to sc transformation, to be of the first order in the order parameter α and of the second order in the order parameter u. Likewise, the continuous pressure dependence of u, flattening at 31.8 GPa, indicates the p-sc to sc transition, corresponding to the second step of the A7 to sc transformation, to be of the second order in the order parameter u.

In this regard, Katzke and Tolédano[37] indicate the first-order character of the A7 to sc phase transition to be imposed by the secondary order parameter represented by the $e_{yz} = e_{xz} = e_{xy}$ spontaneous shear strain components, which transform as the cubic-invariant Γ_5^+ zone-center irreducible representation, whereas the second-order character to be imposed by the primary order parameter represented by the $\eta_1 = \eta_2 = \eta_3$ displacive components, which transform as the symmetry-breaking R_5^+ irreducible representation at the R-corner of the primitive cubic Brillouin zone. Under the evidence that the A7 to sc phase transition in As, like in P, is actually a two-step process, whose steps are indeed separated in pressure, according to Katzke and Tolédano, the first order cubic-invariant primary instability, associated to

the spontaneous shear strain components, corresponds to the A7 ($R\bar{3}m$) to p-sc ($R\bar{3}m$) transition, whereas the second order symmetry-breaking step, associated to the displacive order parameter, corresponds to the p-sc ($R\bar{3}m$) to sc ($Pm\bar{3}m$) transition.

SI-8 Pressure-dependent orbital contributions and electrostatic interactions

Energetically, the crystal structure stability of a system as a function of pressure can be approximated by two main competing contributions, respectively represented by the electronic band energy and by the energy of electrostatic interactions. While the first one is determined by the occupied electronic states and accounts for the orbital driven chemical bonding framework, the second one is essentially dominated by the density in terms of atomic size, charge and packing.[12,38]

Häussermann performed a detailed computational investigation on the *s*, *p* and *d* orbital contributions to the stability of the crystal structures of group 15 elements as a function of pressure, concluding that, whereas the orbital mixing is the driving contribution favoring the formation of open structures in the low pressure regime, electrostatic interactions become dominant with increasing pressure, stabilizing densely packed structures, Specifically, the A7 and sc structures were found to be respectively stabilized by *s*-*p* and *s*-*d* orbital mixing.[12,38]

Although these studies were performed before the experimental report of the p-sc structure in P[6,7] and As, they provide useful insights for the interpretation of our results.

Noticeably, the extent of *s*-*p* orbital mixing decreases with increasing the atomic number in a chemical group. This occurrence is related on one side to the reduction of the *s* orbitals size and to the decrease in their energy due to relativistic effects, and on the other side to the more diffuse character and higher energy of the *p* orbitals, overall weakening the *s*-*p* interaction. The effect of *s*-*p* orbital mixing is thus stronger in P compared to As. Its stabilizing effect on the A7 structure of pnictogens was demonstrated for P and suggested to be possibly relevant also for As by Seo and Hoffmann,[39] before being confirmed by Golden *et al.*[40] Moreover, Golden *et al.* calculated the gap between *s* and *p* contributions to the density of bonding states in As to reduce on compression, actually vanishing at the phase transition as the A7 structure approaches the sc one, and interpreted this result as due to the decrease

of the s-p mixing with pressure and to the onset of metallization.[40,41]

As d orbitals are concerned, it is worth to highlight that, according to Haussermann, s-d mixing increases rapidly with pressure in P while remaining rather low in As and that the extent of the s-d orbital mixing is particularly effective in P, due to the absence of electrons in the $3d$ orbital shell,[38,42] thus determining the onset of the p-sc structure at lower pressure compared to As. The role of empty d orbitals in lowering the A7 to sc transition pressure in P was firstly recognized by Sasaki *et al.*, who calculated the electrostatic contribution to be a key factor in the stabilization of sc P[43] and later reported by Haussermann in the form of s-d mixing.[12,38,42]

In this regard, the contribution of the d orbitals to the sc structure clearly emerges in the electron density of states (DOS) of the sc structures of P (V_{atom}=13.5 Å^3, ~11 GPa) and As (V_{atom}=16.1 Å^3, ~27 GPa) calculated by Häussermann *et al.*[12,38] While the d contribution is higher in P than in As, for both elements the d orbitals contribute to the dramatic increase of the electron DOS at the Fermi level across the A7 to sc phase transition, leading to the bandgap closure and to the semi-metal to metal transition, and in the case of P a significant contribution is appreciable even before the phase transition.[38] Accordingly, the maximum stability pressure of the A7 structure is found to decrease with pressure from As (26.5 GPa, this study) to Sb (8.6 GPa)[44] and Bi (2.52 GPa),[45] consistently with the decrease of s-p orbital mixing. However, due to the extent of the s-d orbital mixing, the high-pressure limit of the A7 structure in P is lower (10.5 GPa) than in As and the stability range of the sc structure extends to much higher pressure in P (~103 GPa)[46,47] than in As (~50 GPa).[48–50] It is worth to remark that, as discovered later, the actual crystal structure of P and As at these calculated pressures is the p-sc structure and not the sc one.[6,7]

Acknowledgements

Thanks are expressed to the following institutions and projects: European Research Council, ERC Advanced Grant No. 670173 PHOSFUN "Phosphorene functionalization: a new platform for advanced multifunctional materials"; CNR, "GreenPhos-alta pressione" and "CHEMPRESS"; Fondazione Cassa di Risparmio di Firenze "HP-PHOTOCHEM"; "NextGenerationEU, Integrated infrastructure initiative in Photonic and Quantum Sciences" I-PHOQS [IR0000016, ID D2B8D520, CUP B53C22001750006]; Made in Italy - Circular and Sustainable (MICS) Extended Partnership, funded by the European Union NextGenerationEU (Piano Nazionale di Ripresa e Resilienza (PNRR) - Missione 4, Componente 2, Investimento 1.3 – D.D. 1551.11-10-2022, PE00000004). The authors acknowledge DESY (Hamburg, Germany), a member of the Helmholtz Association HGF, for the provision of experimental facilities. Parts of this research were carried out at PETRA-III. Acknowledgments are expressed to Hans-Peter Liermann for assistance in using beamline P02.2 (PETRA-III). Beamtime was allocated for proposal I-20190489 EC. The research leading to this result has been supported by the project CALIPSOplus under the Grant Agreement 730872 from the EU Framework Programme for Research and Innovation HORIZON 2020.

Author contributions

M.S-R. synthesized the As single crystals. E.B., A.L., M.C. and M.S-R. conceived and realized the FIB processing of the As single crystals. M.C., M.S-R., D.S., K.D and K.G prepared the DAC, loaded the samples in the DAC and performed the high-pressure XRD experiments. M.M., M.C. and K.D. analyzed the XRD data. All the authors discussed the results. M.C. conceived the experiment and wrote the article.

Competing interests

The authors declare no competing interests.

Supplementary References

(1) Kikegawa, T.; Iwasaki, H. Pressure-Induced Rhombohedral-Simple Cubic Structural Phase Transition in As. *J. Phys. Soc. Jpn.* **1987**, *56*, 3417–3420.

(2) Beister, H. J.; Strössner, K.; Syassen, K. Rhombohedral to simple-cubic phase transition in arsenic under pressure. *Phys. Rev. B* **1990**, *41*, 5535–5543.

(3) Chen, A. L.; Lewis, S. P.; Su, Z.; Yu, P. Y.; Cohen, M. L. Superconductivity in arsenic at high pressures. *Phys. Rev. B* **1992**, *46*, 5523–5527.

(4) Silas, P.; Yates, J. R.; Haynes, P. D. Density-functional investigation of the rhombohedral to simple-cubic phase transition of arsenic. *Phys. Rev. B* **2008**, *78*, 174101.

(5) Hu, Y.; Liang, J.; Xia, Y.; Zhao, C.; Jiang, M.; Ma, J.; Tie, Z.; Jin, Z. 2D Arsenene and Arsenic Materials: Fundamental Properties, Preparation, and Applications. *Small* **2022**, *18*, 2104556.

(6) Scelta, D.; Baldassarre, A.; Serrano-Ruiz, M.; Dziubek, K.; Cairns, A. B.; Peruzzini, M.; Bini, R.; Ceppatelli, M. Interlayer Bond Formation in Black Phosphorus at High Pressure. *Angew. Chem. Int. Ed.* **2017**, *56*, 14135–14140.

(7) Scelta, D.; Baldassarre, A.; Serrano-Ruiz, M.; Dziubek, K.; Cairns, A. B.; Peruzzini, M.; Bini, R.; Ceppatelli, M. The p-sc structure in phosphorus: bringing order to the high pressure phases of group 15 elements. *Chem. Commun.* **2018**, *54*, 10554–10557.

(8) Schirber, J. E.; Van Dyke, J. P. Pressure-Induced "Electron Transition" in As. *Phys. Rev. Lett.* **1971**, *26*, 246–249.

(9) Chang, K. J.; Cohen, M. L. Rhombohedral phase stability of the group-VA elements. *Phys. Rev. B* **1986**, *33*, 7371–7374.

(10) Mattheiss, L. F.; Hamann, D. R.; Weber, W. Structural calculations for bulk As. *Phys. Rev. B* **1986**, *34*, 2190–2198.

(11) da Silva, C. R. S.; Wentzcovitch, R. M. First principles investigation of the A7 to simple cubic transformation in As. *Comput. Mater. Sci.* **1997**, *8*, 219 – 227.

(12) Häussermann, U.; Söderberg, K.; Norrestam, R. Comparative Study of the High-Pressure Behavior of As, Sb, and Bi. *J. Am. Chem. Soc.* **2002**, *124*, 15359–15367.

(13) Durandurdu, M. *Ab initio* simulation of the rhombohedral–to–simple-cubic transition in arsenic. *Phys. Rev. B* **2005**, *72*, 073208.

(14) Feng, W.; Cui, S.; Hu, H.; Liu, H. First-principles study of A7 to simple cubic phase transformation in As. *Physica B* **2007**, *400*, 22–25.

(15) Shang, S.; Wang, Y.; Zhang, H.; Liu, Z.-K. Lattice dynamics and anomalous bonding in rhombohedral As: First-principles supercell method. *Phys. Rev. B* **2007**, *76*, 052301.

(16) Zijlstra, E. S.; Huntemann, N.; Garcia, M. E. Laser-induced solid–solid phase transition in As under pressure: a theoretical prediction. *New. J. Phys.* **2008**, *10*, 033010.

(17) Chan, K. T.; Malone, B. D.; Cohen, M. L. Electron-phonon coupling and superconductivity in arsenic under pressure. *Phys. Rev. B* **2012**, *86*, 094515.

(18) Silas, P. K.; Haynes, P. D.; Yates, J. R. Evolution of the Fermi surface of arsenic through the rhombohedral to simple-cubic phase transition: A Wannier interpolation study. *Phys. Rev. B* **2013**, *88*, 134103.

(19) Seidl, M.; Balázs, G.; Scheer, M. The Chemistry of Yellow Arsenic. *Chem. Rev.* **2019**, *119*, 8406–8434.

(20) Smith, P. M.; Leadbetter, A. J.; Apling, A. J. The structures of orthorhombic and vitreous arsenic. *Philos. Mag.* **1975**, *31*, 57–64.

(21) Wyckoff, R. *Crystal Structures*; Crystal Structures v. 1; Interscience Publishers, 1963.

(22) Li, R.; Han, N.; Cheng, Y.; Huang, W. Pressure-induced metallization of black arsenic. *J. Phys. Condens. Matter* **2019**, *31*, 505501.

(23) Gao, C.; Li, R.; Zhong, M.; Wang, R.; Wang, M.; Lin, C.; Huang, L.; Cheng, Y.; Huang, W. Stability and Phase Transition of Metastable Black Arsenic under High Pressure. *J. Phys. Chem. Lett.* **2020**, *11*, 93–98, PMID: 31802676.

(24) Du, G.; Ke, F.; Han, W.; Chen, B.; Xia, Q.; Kang, J.; Chen, Y. Thermodynamic Origins of Structural Metastability in Two-Dimensional Black Arsenic. *J. Phys. Chem. Lett.* **2023**, *14*, 8676–8683.

(25) Yoshiasa, A.; Tokuda, M.; Misawa, M.; Shimojo, F.; Momma, K.; Miyawaki, R.; Matsubara, S.; Nakatsuka, A.; Sugiyama, K. Natural arsenic with a unique order structure: potential for new quantum materials. *Sci. Rep.* **2019**, *9*, 6275.

(26) Jeavons, A. P.; Saunders, G. A. Single-crystal growth from the vapour and etch pit studies in arsenic. *J. Phys. D Appl. Phys.* **1968**, *1*, 869–873.

(27) Ceppatelli, M.; Scelta, D.; Serrano-Ruiz, M.; Dziubek, K.; Morana, M.; Svitlyk, V.; Garbarino, G.; Poręba, T.; Mezouar, M.; Peruzzini, M.; Bini, R. Single-Bonded Cubic AsN from High-Pressure and High-Temperature Chemical Reactivity of Arsenic and Nitrogen. *Angew. Chem. Int. Ed.* **2022**, *61*, e202114191.

(28) Shen, G.; Wang, Y.; Dewaele, A.; Wu, C.; Fratanduono, D. E.; Eggert, J.; Klotz, S.; Dziubek, K. F.; Loubeyre, P.; Fat'yanov, O. V.; Asimow, P. D.; Mashimo, T.; Wentzcovitch, R. M. M.; other members of the IPPS task group Toward an international practical

pressure scale: A proposal for an IPPS ruby gauge (IPPS-Ruby2020). *High Pressure Res.* **2020**, *40*, 299–314.

(29) Liermann, H.-P.; Konôpková, Z.; Morgenroth, W.; Glazyrin, K.; Bednarčik, J.; McBride, E. E.; Petitgirard, S.; Delitz, J. T.; Wendt, M.; Bican, Y.; Ehnes, A.; Schwark, I.; Rothkirch, A.; Tischer, M.; Heuer, J.; Schulte-Schrepping, H.; Kracht, T.; Franz, H. The Extreme Conditions Beamline P02.2 and the Extreme Conditions Science Infrastructure at PETRA III. *J. Synchrotron Rad.* **2015**, *22*, 908–924.

(30) Rigaku Oxford Diffraction, Oxford, UK. CrysAlisPro Software System. **2021**,

(31) Sheldrick, G. M. *SHELXT* – Integrated space-group and crystal-structure determination. *Acta Crystallogr. Sect. A* **2015**, *71*, 3–8.

(32) Sheldrick, G. M. Crystal structure refinement with *SHELXL*. *Acta Crystallogr. Sect. C* **2015**, *71*, 3–8.

(33) Angel, R. J.; Mazzucchelli, M. L.; Baratelli, L.; Schweinle, C. F.; Balić-Žunić, T.; Gonzalez-Platas, J.; Alvaro, M. Uncertainties of recalculated bond lengths, angles and polyhedral volumes as implemented in the *Crystal Palace* program for parametric crystal structure analysis. *Acta Crystallogr. Sect. A* **2025**, *81*, 202–210.

(34) Prescher, C.; Prakapenka, V. B. DIOPTAS: a program for reduction of two-dimensional X-ray diffraction data and data exploration. *High Pressure Res.* **2015**, *35*, 223–230.

(35) Schwarzenbach, D.; Abrahams, S. C.; Flack, H. D.; Gonschorek, W.; Hahn, T.; Huml, K.; Marsh, R. E.; Prince, E.; Robertson, B. E.; Rollett, J. S.; Wilson, A. J. C. Statistical descriptors in crystallography: Report of the IUCr Subcommittee on Statistical Descriptors. *Acta Crystallogr. Sect. A* **1989**, *45*, 63–75.

(36) Needs, R. J.; Martin, R. M.; Nielsen, O. H. Total-energy calculations of the structural properties of the group-V element arsenic. *Phys. Rev. B* **1986**, *33*, 3778–3784.

(37) Katzke, H.; Tolédano, P. Displacive mechanisms and order-parameter symmetries for the A7-incommensurate-bcc sequences of high-pressure reconstructive phase transitions in Group Va elements. *Phys. Rev. B* **2008**, *77*, 024109.

(38) Häussermann, U. High-Pressure Structural Trends of Group 15 Elements: Simple Packed Structures versus Complex Host–Guest Arrangements. *Chem. Eur. J.* **2003**, *9*, 1471–1478.

(39) Seo, D.-K.; Hoffmann, R. What Determines the Structures of the Group 15 Elements? *J. Solid State Chem.* **1999**, *147*, 26–37.

(40) Golden, J. C.; Ho, V.; Lubchenko, V. The chemical bond as an emergent phenomenon. *J. Chem. Phys.* **2017**, *146*, 174502.

(41) A. Papoian, G.; Hoffmann, R. Hypervalent Bonding in One, Two, and Three Dimensions: Extending the Zintl–Klemm Concept to Nonclassical Electron-Rich Networks. *Angew. Chem. Int. Ed.* **2000**, *39*, 2408–2448.

(42) Mikhaylushkin, A. S.; Simak, S. I.; Johansson, B.; Häussermann, U. High-pressure behavior of phosphorus from first principles calculations. *Phys. Rev. B* **2007**, *76*, 092103.

(43) Sasaki, T.; Shindo, K.; Niizeki, K.; Morita, A. Structural Stability and Band Structure of the Metallic Phase of Phosphorus. *J. Phys. Soc. Jpn.* **1988**, *57*, 978–987.

(44) Schiferl, D.; Cromer, D. T.; Jamieson, J. C. Structure determinations on Sb up to 85×10^2 MPa. *Acta Crystallogr. Sect. B* **1981**, *37*, 807–810.

(45) Getting, I. C. New determination of the bismuth I-II equilibrium pressure: a proposed modification to the practical pressure scale. *Metrologia* **1998**, *35*, 119–132.

(46) Akahama, Y.; Kobayashi, M.; Kawamura, H. Simple-cubic–simple-hexagonal transition in phosphorus under pressure. *Phys. Rev. B* **1999**, *59*, 8520–8525.

(47) Sugimoto, T.; Akahama, Y.; Fujihisa, H.; Ozawa, Y.; Fukui, H.; Hirao, N.; Ohishi, Y. Identification of superlattice structure $cI16$ in the P-VI phase of phosphorus at 340 GPa and room temperature via x-ray diffraction. *Phys. Rev. B* **2012**, *86*, 024109.

(48) Greene, R. G.; Luo, H.; Ruoff, A. L. bcc arsenic at 111 GPa: An x-ray structural study. *Phys. Rev. B* **1995**, *51*, 597–600.

(49) Degtyareva, O.; McMahon, M. I.; Nelmes, R. J. High-pressure structural studies of group-15 elements. *High Pressure Res.* **2004**, *24*, 319–356.

(50) Akahama, Y.; Kamiue, K.; Okawa, N.; Kawaguchi, S.; Hirao, N.; Ohishi, Y. Volume compression of period 4 elements: Zn, Ge, As, and Se above 200 GPa: Ordering of atomic volume by atomic number. *J. Appl. Phys.* **2021**, *129*, 025901 1–8.
